# Novel “Superspreader” Bacteriophages Promote Horizontal Gene Transfer by Transformation

**DOI:** 10.1128/mBio.02115-16

**Published:** 2017-01-17

**Authors:** Eric C. Keen, Valery V. Bliskovsky, Francisco Malagon, James D. Baker, Jeffrey S. Prince, James S. Klaus, Sankar L. Adhya

**Affiliations:** a Department of Biology, University of Miami, Coral Gables, Florida, USA; b Laboratory of Molecular Biology, Center for Cancer Research, National Cancer Institute, National Institutes of Health, Bethesda, Maryland, USA; c Laboratory of Cancer Biology and Genetics, Center for Cancer Research, National Cancer Institute, National Institutes of Health, Bethesda, Maryland, USA; d Henry M. Jackson Foundation, Biological Defense Research Directorate, Naval Medical Research Center, Fort Detrick, Maryland, USA; e Department of Geological Sciences, University of Miami, Coral Gables, Florida, USA; Yale School of Medicine

## Abstract

Bacteriophages infect an estimated 10^23^ to 10^25^ bacterial cells each second, many of which carry physiologically relevant plasmids (e.g., those encoding antibiotic resistance). However, even though phage-plasmid interactions occur on a massive scale and have potentially significant evolutionary, ecological, and biomedical implications, plasmid fate upon phage infection and lysis has not been investigated to date. Here we show that a subset of the natural lytic phage population, which we dub “superspreaders,” releases substantial amounts of intact, transformable plasmid DNA upon lysis, thereby promoting horizontal gene transfer by transformation. Two novel *Escherichia coli* phage superspreaders, SUSP1 and SUSP2, liberated four evolutionarily distinct plasmids with equal efficiency, including two close relatives of prominent antibiotic resistance vectors in natural environments. SUSP2 also mediated the extensive lateral transfer of antibiotic resistance in unbiased communities of soil bacteria from Maryland and Wyoming. Furthermore, the addition of SUSP2 to cocultures of kanamycin-resistant *E. coli* and kanamycin-sensitive *Bacillus* sp. bacteria resulted in roughly 1,000-fold more kanamycin-resistant *Bacillus* sp. bacteria than arose in phage-free controls. Unlike many other lytic phages, neither SUSP1 nor SUSP2 encodes homologs to known hydrolytic endonucleases, suggesting a simple potential mechanism underlying the superspreading phenotype. Consistent with this model, the deletion of endonuclease IV and the nucleoid-disrupting protein ndd from coliphage T4, a phage known to extensively degrade chromosomal DNA, significantly increased its ability to promote plasmid transformation. Taken together, our results suggest that phage superspreaders may play key roles in microbial evolution and ecology but should be avoided in phage therapy and other medical applications.

## IMPORTANCE

Bacteriophages (phages), viruses that infect bacteria, are the planet’s most numerous biological entities and kill vast numbers of bacteria in natural environments. Many of these bacteria carry plasmids, extrachromosomal DNA elements that frequently encode antibiotic resistance. However, it is largely unknown whether plasmids are destroyed during phage infection or released intact upon phage lysis, whereupon their encoded resistance could be acquired and manifested by other bacteria (transformation). Because phages are being developed to combat antibiotic-resistant bacteria and because transformation is a principal form of horizontal gene transfer, this question has important implications for biomedicine and microbial evolution alike. Here we report the isolation and characterization of two novel *Escherichia coli* phages, dubbed “superspreaders,” that promote extensive plasmid transformation and efficiently disperse antibiotic resistance genes. Our work suggests that phage superspreaders are not suitable for use in medicine but may help drive bacterial evolution in natural environments.

## INTRODUCTION

Bacteriophages, viruses that infect bacteria, and plasmids, extrachromosomal bacterial genetic elements, are ubiquitous in natural environments. Although phages are thought to kill roughly one-quarter of the planet’s bacteria every day (1), many of which carry plasmids (2), it is unclear whether bacterial plasmids are degraded during phage infection or released intact upon phage lysis. Because the uptake of exogenous DNA (transformation) is a principal form of horizontal gene transfer (3), because phages are actively being developed as treatments for antibiotic-resistant bacterial infections (1), and because antibiotic resistance genes are frequently located on plasmids (4, 5), the fate of plasmids upon phage infection and lysis has potentially significant evolutionary, ecological, and biomedical implications.

Phages’ ability to physically shuttle DNA between bacteria (transduction) has been recognized for >60 years (6), and numerous phages capable of transducing plasmids, including those encoding antibiotic resistance, have been previously described (7). However, phages’ potential relevance for bacterial transformation has received considerably less attention, a fact underscored by several recent reviews’ exclusive focus on transduction in discussing phage-mediated gene transfer (8–10). Although phage infection and lysis are presumed to significantly enrich pools of extracellular DNA in natural environments (11), many fundamental questions, including the variability in different phages’ capacity to release intact host DNA, the distinguishing mechanism(s) associated with such release, and the transformability of phage-released host DNA, have not been investigated to date.

In the present study, we explored these and similar questions by isolating and evaluating a library of novel lytic phages that infected a common host, *Escherichia coli* strain MG1655. We developed simple lysis-and-transformation assays by which we quantified the abilities of individual phage isolates to promote the transformation of various plasmids. In parallel, we employed several different forms of quantitative PCR to measure the amount of plasmid DNA released intact upon phage lysis. Through these assays, we identified two novel coliphages that, in contrast to the majority of our library, promoted extensive plasmid transformation *in vitro*. We sequenced the complete genomes of these two phages and posited a mechanism for their activity, which we then evaluated experimentally. Finally, and most importantly, we assessed our two phages’ ability to promote plasmid transformation and disseminate antibiotic resistance under coculture conditions approximating those *in situ*.

## RESULTS

### Phages vary greatly in their ability to promote plasmid transformation.

We devised a simple functional assay to quantify the extent to which different phages mediate plasmid transformation in a common host. Briefly, equal numbers of *E. coli* MG1655 (12) cells bearing ampicillin resistance plasmid pBAD24 (13) (MG/pBAD24) were infected and lysed by various coliphages at a multiplicity of infection (MOI) of >10 to ensure equal bacterial killing, followed by DNA extraction from each lysate, transformation into chemically competent (14), antibiotic-sensitive *E. coli* MG1655, and plating on selective media (see Text S1 in the supplemental material). Using this assay, we screened two model lytic coliphages, T4 and T7, and 20 novel lytic coliphages isolated from soil, water, and fecal samples in the vicinity of Miami, FL, and Washington, DC.

10.1128/mBio.02115-16.1 TEXT S1  Complete materials and methods used in this study. Download Text S1, DOCX file, 0.03 MB. Copyright © 2017 Keen et al. 2017 Keen et al. This content is distributed under the terms of the Creative Commons Attribution 4.0 International license.

**FIG 1:**
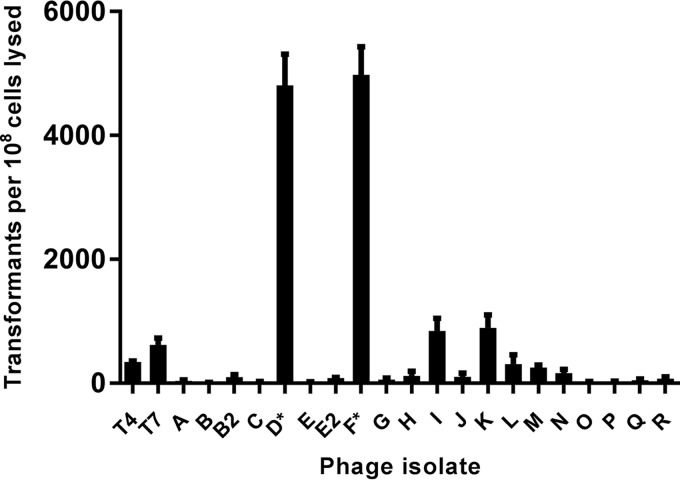
Two novel lytic coliphages promote extensive plasmid DNA transformation. Data represent the numbers of antibiotic-resistant transformants obtained from DNA extracted from various phages’ lysates of ~10^8^ *E. coli* MG1655 cells bearing ampicillin resistance plasmid pBAD24. Because known—but slightly varying—numbers of *E. coli* MG1655 cells were used in different replicates, raw transformant counts were normalized to the per-10^8^-cells-lysed standard. Error bars represent the standard error of the mean of eight independent biological replicates of isolates D* and F* and three independent biological replicates of all other phages. Phage isolates D* and F* were subsequently renamed SUSP1 and SUSP2, respectively (see text).

Infection and lysis by different phages resulted in substantially different numbers of antibiotic-resistant transformants (Fig. 1). Although the majority of phage lysates yielded <100 transformants per 10^8^ cells lysed, two particular isolates were roughly 50-fold more efficient at promoting plasmid transformation. Because this result—a small proportion of the population playing an outsized role in transmission—was evocative of a similar phenomenon in epidemiology (15), we chose the term “superspreaders” to describe these two isolates and designated them *Escherichia* phages SUSP1 and SUSP2.

### Phage-mediated plasmid transformation is not sequence specific.

**FIG 2:**
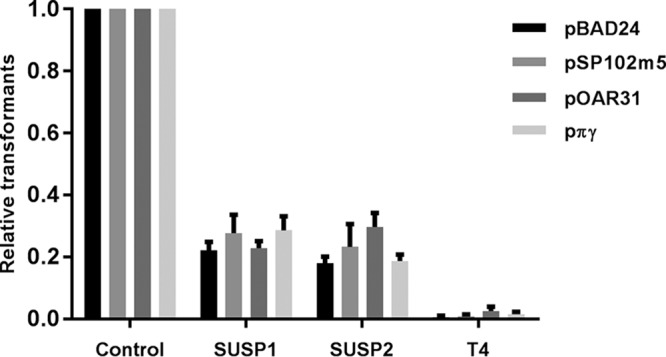
Phages SUSP1 and SUSP2, but not T4, promote the transformation of four evolutionarily distinct plasmids. Data represent the yields of antibiotic-resistant transformants obtained from DNA extracted from lysates in which ~10^8^ *E. coli* MG1655 cells bearing one of four resistance plasmids (pBAD24, pSP102m5, pOAR31, or pπγ) were lysed by phage SUSP1, SUSP2, or T4. Because known—but slightly varying—numbers of *E. coli* MG1655 cells were used in different replicates, raw transformant counts were normalized to the per-10^8^-cells-lysed standard. Data represent the mean proportions of the control (transformation of plasmid DNA extracted with a commercial kit from the same number of bacterial cells as were lysed by phage) for each plasmid type. Error bars represent the standard error of the mean of three independent biological replicates.

We next assessed the ability of SUSP1 and SUSP2 to promote the transfer of other plasmids besides pBAD24. We created *E. coli* MG1655 strains bearing one of three evolutionarily dissimilar plasmids—pSP102m5, pOAR31, and pπγ (16, 17)—and infected those strains and MG/pBAD24 with SUSP1, SUSP2, or T4, followed by DNA extraction and transformation as described above . Like many lytic phages, T4 expresses multiple hydrolytic endonucleases during infection (18) and was thus used as a control for phage-mediated DNA degradation in these and subsequent experiments. Lysis of all four plasmid-bearing strains by SUSP1 and SUSP2 yielded substantial and proportionally similar numbers of transformants, while T4 yielded >10-fold fewer transformants for all plasmids tested (Fig. 2). These results strongly suggest that the ability of superspreader phages to promote plasmid transformation is generalized, not sequence specific.

### Phage-mediated plasmid transformation is distinct from transduction.

To confirm that our lysis-and-transformation assays were truly reflecting phages’ liberation of plasmid DNA directly upon lysis, rather than phage packaging of plasmid DNA (transduction) and its subsequent release upon capsid disruption during DNA extraction, we pelleted free phage particles in SUSP2 lysates of MG/pBAD24 by centrifugation and transformed competent cells with DNA purified from the resulting supernatants. Although centrifugation reduced free phage titers by roughly 1,000-fold, similar numbers of ampicillin-resistant transformants were obtained from centrifuged and uncentrifuged lysates (see Fig. S1), thereby excluding transduction as a significant contributor to our results.

10.1128/mBio.02115-16.2 FIGURE S1  Removal of SUSP2 phage particles via centrifugation does not affect transformant yields. Data represent the number of antibiotic-resistant transformants (black bars; left *y* axis) and free phage titers (gray bars; right *y* axis) obtained from SUSP2 lysates of ~10^8^ *E. coli* MG1655 cells bearing ampicillin resistance plasmid pBAD24. Spun lysates were centrifuged for 1 h at 21,000 × *g* prior to DNA extraction; unspun lysates were not centrifuged. Because known—but slightly varying—numbers of *E. coli* MG1655 cells were used in different replicates, raw transformant counts were normalized to the per-10^8^-cells-lysed standard. Phage titers were determined by plaque assay. Error bars represent the standard error of the mean of three independent biological replicates. The asterisk denotes statistical significance at a standard of *P* < 0.05 (Mann-Whitney U test); ns denotes statistical insignificance. Download Figure S1, TIF file, 0.1 MB. Copyright © 2017 Keen et al. 2017 Keen et al. This content is distributed under the terms of the Creative Commons Attribution 4.0 International license.

### Phage-mediated plasmid transformation does not result from “lysis from without.”

To control for varying replication kinetics among the coliphages in our library, our lysis-and-transformation assays involved saturating doses of phages (MOI > 10) to eliminate nearly all susceptible bacterial cells with a single round of infection. However, massive phage adsorption can, under certain circumstances, fatally destabilize the bacterial outer membrane and lead to host cell death via “lysis from without” even in the absence of productive infection (“lysis from within”) (19). To confirm that superspreader phage activity was not MOI dependent, we conducted additional lysis-and-transformation assays with 100-fold fewer SUSP2 particles per initial infection (MOI < 1). Considerably more antibiotic-resistant bacterial transformants resulted from these low-MOI infections (see Fig. S2), a result incompatible with lysis from without but one consistent with increased replication of uninfected plasmid donors prior to eventual catch-up by SUSP2 (i.e., more plasmid-bearing cells being lysed overall). Thus, the superspreader phenotype is not an artifact associated with high MOIs but rather a characteristic of productive SUSP1 and SUSP2 infection.

10.1128/mBio.02115-16.3 FIGURE S2  Phage SUSP2-mediated plasmid transformation is not limited to high-MOI conditions. Data represent antibiotic-resistant transformant yields from DNA extracted from lysates in which ~10^8^ *E. coli* MG1655 cells bearing plasmid pBAD24 were lysed by phage SUSP2 at an MOI of >10 (left) or <1 (right). Because known—but slightly varying—numbers of *E. coli* MG1655 cells were used in different replicates, raw transformant counts were normalized to the per-10^8^-cells-lysed standard. Horizontal lines within each cluster of data points represent the mean values of three independent biological replicates. ns denotes statistical insignificance (Mann-Whitney U test). Download Figure S2, TIF file, 0.3 MB. Copyright © 2017 Keen et al. 2017 Keen et al. This content is distributed under the terms of the Creative Commons Attribution 4.0 International license.

### Superspreader phages release greater quantities of intact plasmid DNA.

We next quantified phages’ differential release of plasmid DNA in a transformation-independent manner by conducting digital droplet PCR (ddPCR; Bio-Rad) with DNA extracted from SUSP2 and T4 lysates of MG/pBAD24. We amplified 130-bp fragments from pBAD24 and from the *E. coli* chromosomal gene *rpoS*, a product size within the recommended range (75 to 200 bp) for ddPCR. Consistent with previous reports that T4 extensively hydrolyzes host chromosomal DNA during infection (18, 20), SUSP2 lysates contained >30-fold more 130-bp *rpoS* copies than did T4 lysates (see Fig. S3). Unexpectedly, however, SUSP2 and T4 lysates contained nearly identical 130-bp pBAD24 copy numbers (see Fig. S3), suggesting that T4 does not degrade plasmid DNA to nucleotides as it does chromosomal DNA (20). We further explored this issue by combining long-range Phusion PCR (Thermo Fisher) with the TapeStation platform (Agilent Technologies) to amplify and quantify nearly the entire pBAD24 plasmid (4,480 of 4,542 bp or 98.6%), a product far too large to assess by ddPCR or by conventional quantitative PCR. In contrast to the ddPCR results but consistent with our initial transformation screening (Fig. 1), SUSP2 lysates of MG/pBAD24 yielded approximately 10-fold greater band intensity (i.e., whole-plasmid copies) than did corresponding T4 lysates (see Fig. S4). Accordingly, superspreading phages’ superior capacity to facilitate transformation is explained most parsimoniously by their superior release of physically intact plasmids.

10.1128/mBio.02115-16.4 FIGURE S3  Phages SUSP2 and T4 do not extensively degrade plasmid DNA to <130-bp fragments. DNA was extracted from SUSP2 and T4 lysates of ~10^8^ *E. coli* MG1655 cells bearing ampicillin resistance plasmid pBAD24 and assessed via ddPCR. Data represent the copy numbers of 130-bp fragments from pBAD24 (black circles) and from the *E. coli* chromosomal gene *rpoS* (gray squares) as measured by ddPCR. Template-free negative controls yielded <10^3^ copies for all replicates, indicating minimal basal contamination. Because known—but slightly varying—numbers of *E. coli* MG1655 cells were used in different replicates, raw transformant counts were normalized to the per-10^8^-cells-lysed standard. The control represents transformation of plasmid DNA extracted with a commercial kit from the same number of bacterial cells as were lysed by phage. Horizontal lines within each cluster of data points represent the mean values of at least four independent biological replicates. The asterisk denotes statistical significance at a standard of *P* < 0.05 (Mann-Whitney U test); ns denotes statistical insignificance. Download Figure S3, TIF file, 0.2 MB. Copyright © 2017 Keen et al. 2017 Keen et al. This content is distributed under the terms of the Creative Commons Attribution 4.0 International license.

10.1128/mBio.02115-16.5 FIGURE S4  Phage SUSP2 releases significantly more intact plasmid pBAD24 DNA than does T4. DNA was extracted from SUSP2 and T4 lysates of ~10^8^ *E. coli* MG1655 cells bearing ampicillin resistance plasmid pBAD24, amplified by long-range Phusion PCR, and quantified via TapeStation analysis. Relative band intensity represents the relative abundance of intact, whole plasmid pBAD24 (~4.5 kb) copies in SUSP2 lysates (lane C1) and T4 lysates (lane D1) of approximately 10^8^ *E. coli* MG1655 cells bearing pBAD24. The positive control (lane B1) represents Phusion PCR amplification of plasmid DNA extracted with a commercial kit from the same number of bacterial cells as were lysed by phage. The negative control (lane E1) represents a no-template control for Phusion PCR. Download Figure S4, TIF file, 0.5 MB. Copyright © 2017 Keen et al. 2017 Keen et al. This content is distributed under the terms of the Creative Commons Attribution 4.0 International license.

### Phage superspreaders do not encode homologs to known hydrolytic endonucleases.

Next, we visualized the morphologies of SUSP1 and SUSP2 by electron microscopy and sequenced their entire genomes (see Fig. S5). Both SUSP1 and SUSP2 are members of the family *Myoviridae* and form clear plaques (see Fig. S6); possess genomes of 90.8 and 88.7 kb, respectively; and are most closely related to *Escherichia* phage EC6 (21), *Citrobacter* phage Moogle (22), and other Felix O1-like viruses (see Table S1). Because both SUSP1 and SUSP2 share <90% whole-genome homology with all other published phage sequences and encode a substantial number of hypothetical proteins with unknown biological functions, we considered the possibility that these phages actively produce some type of DNA-protecting factor(s) during infection. However, we also observed that SUSP1 and SUSP2, like Felix O1 (23) but unlike T4 and many other lytic phages (18), do not encode any gene products resembling known hydrolytic endonucleases, thereby suggesting a simpler, if passive, mechanism by which host DNA and plasmids could conceivably survive phage infection intact.

10.1128/mBio.02115-16.6 FIGURE S5  Phages SUSP1 and SUSP2 display extensive synteny. Genome maps of phages SUSP1 and SUSP2 are shown. Green and red bars depict enzymes associated with nucleotide metabolism and DNA replication, respectively. Dark blue and pink bars depict structural proteins and assembly-related enzymes, respectively. Yellow bars represent lysis-related genes, and light green bars (innermost circles) represent genes encoding tRNAs. Download Figure S5, TIF file, 2.8 MB. Copyright © 2017 Keen et al. 2017 Keen et al. This content is distributed under the terms of the Creative Commons Attribution 4.0 International license.

10.1128/mBio.02115-16.7 FIGURE S6  Phages SUSP1 and SUSP2 are members of the family *Myoviridae*. Phages SUSP1 (a) and SUSP2 (b) were negatively stained with 2% uranyl acetate, and their morphologies were visualized via transmission electron microscopy. Scale bars, 50 nm. Inset images show plaque formation on bacterial lawns by phages SUSP1 and SUSP2. Inset scale bars, 25 μm. Download Figure S6, TIF file, 61.7 MB. Copyright © 2017 Keen et al. 2017 Keen et al. This content is distributed under the terms of the Creative Commons Attribution 4.0 International license.

10.1128/mBio.02115-16.9 TABLE S1  The 15 extant phage genome sequences most closely related to *Escherichia* phage SUSP1, as determined by whole-genome nucleotide BLAST (MegaBLAST) analysis. Download Table S1, DOCX file, 0.02 MB. Copyright © 2017 Keen et al. 2017 Keen et al. This content is distributed under the terms of the Creative Commons Attribution 4.0 International license.

### Unrelated phages lacking suites of hydrolytic endonucleases also promote plasmid transformation.

**FIG 3:**
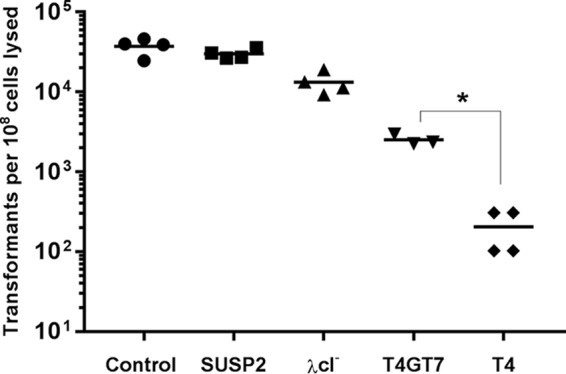
Certain non-SUSP phages also promote significant plasmid transformation. Data represent the yields of antibiotic-resistant transformants obtained from DNA extracted from lysates in which ~10^8^ *E. coli* Le392 cells bearing ampicillin resistance plasmid pBAD24 were lysed by phage SUSP2 (squares), λ*c*I^−^ (triangles), T4GT7 (inverted triangles), or wild-type T4 (diamonds). Because known—but slightly varying—numbers of *E. coli* MG1655 cells were used in different replicates, raw transformant counts were normalized to the per-10^8^-cells-lysed standard. The control represents transformation of plasmid DNA extracted with a commercial kit from the same number of bacterial cells as were lysed by phage. Horizontal lines within each cluster of data points represent the mean values of at least three independent biological replicates. The asterisk denotes statistical significance at a standard of *P* < 0.05 (Mann-Whitney U test).

To evaluate this question experimentally and to confirm that phage superspreading is not limited to a specific plasmid donor or recipient strain (i.e., *E. coli* MG1655), we transformed plasmid pBAD24 into *E. coli* Le392 (Le/pBAD24) and produced lysates thereof with phages SUSP2, T4, λ*c*I^−^, and T4GT7 (see Table S2). Phage λ*c*I^−^ is a lytic derivative of the canonical temperate phage λ, which encodes a single-stranded-DNA-dependent endonuclease (24) but does not disrupt bacterial DNA upon infection (25). Phage T4GT7 is a mutant of T4 that is, unlike wild-type T4, capable of efficient generalized transduction (26). In this second set of lysis-and-transformation assays, in which DNA was extracted from phage lysates of Le/pBAD24 and transformed into wild-type *E. coli* DH5α, SUSP2 manifested even greater superspreading efficiency than in *E. coli* MG1655, strongly suggesting that phage-mediated plasmid transfer is not strain specific (Fig. 3). Moreover, in these same assays, both λ*c*I^−^ and T4GT7 promoted >10-fold more plasmid transformation than did wild-type T4 (Fig. 3).

10.1128/mBio.02115-16.10 TABLE S2  Descriptions of the bacteria, phages, plasmids, and primers used in this study. Download Table S2, DOCX file, 0.02 MB. Copyright © 2017 Keen et al. 2017 Keen et al. This content is distributed under the terms of the Creative Commons Attribution 4.0 International license.

Despite its significantly enhanced rate of plasmid transfer, T4GT7 differs from wild-type T4 in only 7 of 288 total genes; 2 genes involved in the incorporation of 5-hydroxymethylcytosine into T4 DNA (*g42*, *g56*), a gene that blocks transcription of DNA with unmodified cytosines (*alc*), 2 membrane-related genes (*ac* and *rIIB*), and 2 genes (*denB* and *ndd*) whose products (endonuclease IV and a DNA-binding protein, respectively) are involved in DNA degradation (27, 28). Of these, *denB* and *ndd* are the most plausibly related to plasmid fate during infection, and the deletion of both is required for efficient T4GT7 transduction (28). The failure of T4GT7 to fully recapitulate a superspreader phenotype may result from the unabridged activity of genes such as *denA*, whose product, endonuclease II, also strongly contributes to host chromosome degradation (20). Conversely, λ*c*I^−^ lacks T4-like endonucleases altogether, a functional commonality with SUSP1 and SUSP2 despite belonging to a different taxonomic family (*Siphoviridae*), encoding a considerably smaller genome (48.5 kb), and sharing no regions of >25 bp with significant sequence similarity. Taken together, our data are thus most consistent with a model in which the absence of plasmid-degrading endonucleases, rather than the DNA-protecting activity of a specific gene product, mediates the survival of plasmid DNA during superspreader phage infection. However, considering the substantial proportion of SUSP1 and SUSP2 gene products with unknown functions, further research is required to fully clarify the mechanism(s) underlying phage-mediated plasmid transformation.

### Phage superspreaders disseminate antibiotic resistance in mixed populations of soil bacteria.

**FIG 4:**
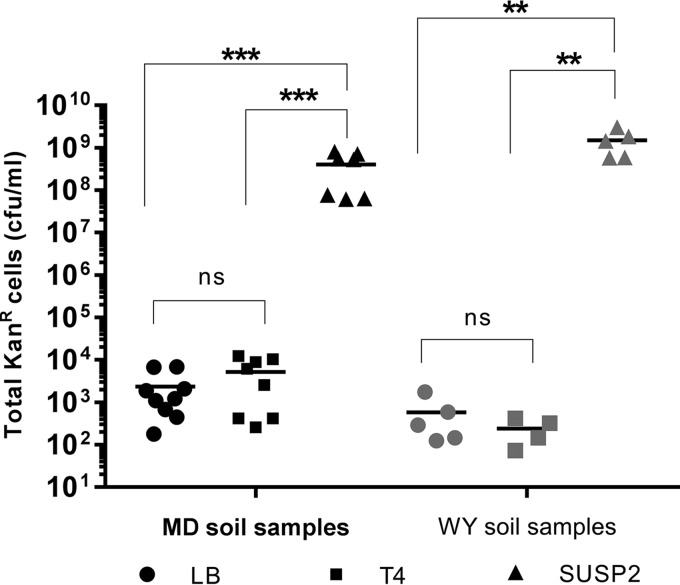
Phage SUSP2 promotes plasmid transformation and antibiotic resistance transfer in heterogeneous populations of soil bacteria. Data represent the total number of kanamycin-resistant cells in cultures of soil bacteria following overnight incubation in LB medium (circles), in T4 lysates of *E. coli* MG1655 bearing kanamycin resistance plasmid pπγ (squares), or in SUSP2 lysates of the same (triangles). Soil samples were collected from three sites in Maryland (black data points) and two sites in Wyoming (gray data points). Phage lysates were confirmed to be free of bacteria prior to inoculation via plating on media containing 50 µg/ml kanamycin (no colony formation) and by overnight incubation in sterile LB medium (no resulting turbidity). Horizontal lines within each cluster of data points represent the mean values of at least four independent biological replicates. Three asterisks denote statistical significance at a standard of *P* < 0.001, two asterisks denote statistical significance at a standard of *P* < 0.01, and ns denotes statistical insignificance (Mann-Whitney U test).

The above-mentioned transformation experiments were all conducted with long-standing laboratory strains of *E. coli* that had been made chemically competent prior to receiving purified DNA. We therefore sought to determine whether phage superspreaders could promote transformation in a more natural context. We collected soil samples from multiple sites in Bethesda, MD, and Cheyenne, WY, and extracted bacteria thereof via simple washing. Identical aliquots of soil bacteria from each site were grown as overnight cultures in LB medium and in cell-free SUSP2 and T4 lysates of MG/pπγ, which were subsequently plated to quantify the proportion of antibiotic-resistant transformants in each culture. Remarkably, communities of soil bacteria from both Maryland and Wyoming displayed >1,000-fold more resistance when propagated overnight in SUSP2 lysates than when propagated in either T4 lysates or in LB medium (Fig. 4), suggesting that superspreader phages can promote plasmid transformation—and the acquisition of antibiotic resistance—in heterogeneous populations of soil bacteria and in the absence of artificial transformation protocols. Because certain environmental bacteria manifest conditional competence under nonlaboratory conditions (29) and because the overwhelming majority of environmental bacteria are unculturable by traditional techniques (30, 31), the pool of potential plasmid recipients would likely be greater still *in situ*.

### *E. coli*-specific phage superspreaders disperse antibiotic resistance to *Bacillus* sp. in coculture.

**FIG 5:**
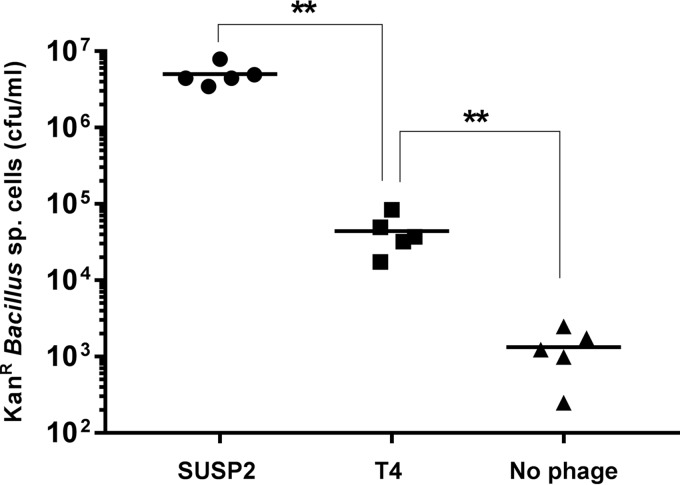
Phage SUSP2 promotes the acquisition of kanamycin resistance by *Bacillus* strain WY10 when cocultured with kanamycin-resistant *E. coli*. A total of 10^8^ WY10 cells and 10^8^ *E. coli* MG1655 cells bearing kanamycin resistance plasmid pπγ were cocultured with 10^7^ SUSP2 phage (circles), 10^7^ T4 phage (squares), or no added phage (triangles). Wild-type WY10 is sensitive to kanamycin and has a non-*E. coli* colony morphotype. Data represent the number of kanamycin-resistant WY10 cells following overnight coculture. No kanamycin-resistant *Bacillus* cells were detected upon coculture with wild-type (plasmid-free) *E. coli* MG1655. Horizontal lines within each cluster of data points represent the mean values of five independent biological replicates. Two asterisks denote statistical significance at a standard of *P* < 0.01 (Mann-Whitney U test).

In the above-mentioned natural transformation experiments, we observed an abundant, non-*E. coli* colony morphotype in platings of Wyoming soil bacteria that had acquired kanamycin resistance via SUSP2 lysis of MG/pπγ. We plated diluted soil aliquots directly onto nonselective media and isolated the wild-type strain of this bacterium, dubbed WY10, which was kanamycin sensitive and refractory to SUSP2 infection (data not shown). 16S rRNA sequencing established WY10 within the Gram-positive genus *Bacillus*, whose species are ubiquitous in soil and frequently exhibit natural competence (32). Subsequent whole-genome sequencing identified WY10 as a novel *Bacillus* strain most closely related (92% identity over 461.7 kb) to *B. simplex* strain SH-B26 (GenBank accession no. CP011008). To further assess the ability of SUSP2 to disseminate antibiotic resistance genes to soil bacteria under conditions approximating those in natural environments, we cocultured WY10 and MG/pπγ with either SUSP2 or T4 (MOI of 0.1) and quantified the resulting proportions of kanamycin-resistant WY10 following overnight incubation. Consistent with our original natural transformation assays (Fig. 4) and with previous reports that certain *E. coli* plasmids can also replicate in *Bacillus* (25), WY10 populations manifested approximately 100-fold more kanamycin resistance when cocultured with SUSP2 than when cocultured with T4 (Fig. 5). Minimal resistance (>1,000-fold lower than with SUSP2) was observed when MG/pπγ and WY10 were cocultured in the absence of phage, and no resistance was detected when wild-type *E. coli* MG1655 was substituted for MG/pπγ (Fig. 5). As expected, plasmid pπγ was repeatedly reisolated from randomly selected WY10 colonies that grew on plates containing kanamycin (see Fig. S7). These results underscore the abilities of superspreader phages to promote interspecific plasmid transformation and to facilitate the acquisition of antibiotic resistance by bacteria only distantly related to their own hosts.

10.1128/mBio.02115-16.8 FIGURE S7  Plasmid pπγ is the source of kanamycin resistance in *Bacillus* strain WY10 following coculture. *E. coli* MG1655 (MG/pπγ), kanamycin-sensitive *Bacillus* strain WY10, and phage SUSP2 were cocultured overnight and plated on selective media. Plasmid DNA was extracted from control MG/pπγ cells (lane B1) and from two randomly selected kanamycin-resistant isolates of *Bacillus* strain WY10 (lanes D1 and E1) and then analyzed via the TapeStation platform. Wild-type WY10 is kanamycin sensitive and does not contain pπγ (lane C1). Identical banding patterns confirm that the kanamycin-resistant WY10 phenotype obtained upon coculture with kanamycin-resistant *E. coli* (Fig. 5) is a result of the natural transformation of plasmid pπγ. Download Figure S7, TIF file, 0.4 MB. Copyright © 2017 Keen et al. 2017 Keen et al. This content is distributed under the terms of the Creative Commons Attribution 4.0 International license.

## DISCUSSION

Although bacteriophages have long been known to contribute to horizontal gene transfer (6), previous investigations have focused almost exclusively on certain phages’ capacity for transduction (8–10). Here we describe a distinct form of phage-mediated horizontal gene transfer, one characterized by the release of intact bacterial DNA upon lysis, and report the isolation and preliminary characterization of two novel *Escherichia* phages, SUSP1 and SUSP2, whose distinguishing transformation-promoting phenotype has not been previously described. Despite the scope of transduction in natural environments (33), even the most promiscuous transducing phage can only disseminate DNA among bacteria it can infect. In contrast, our results suggest that phage superspreaders can exert effects that extend well beyond their own host ranges. For example, our natural transformation assays (Fig. 4) implicitly assessed plasmid uptake by bacteria refractory to SUSP2, since SUSP2 virions were not removed or inactivated prior to the inoculation of lysates with soil bacteria. Subsequently, we explicitly observed that SUSP2 infection and lysis of kanamycin-resistant *E. coli* promoted the acquisition of resistance by a soil-derived *Bacillus* strain, WY10, that was uninfectable by SUSP2 (Fig. 5). Both SUSP1 and SUSP2 efficiently liberated two broad-host-range plasmids, pOAR31 and pπγ, whose environmental counterparts are prominent vectors of virulence factors and antibiotic resistance genes (5, 34, 35). Ultimately, the biological relevance of superspreading phages is likely to be principally regulated by the host ranges of their liberated plasmids, by the relative abundance of naturally competent bacteria compatible with those plasmids, and by factors that influence the persistence of DNA in natural environments.

Since the virosphere comprises an estimated 10^31^ phages (36), of which we surveyed 20, it is exceedingly implausible that SUSP1 and SUSP2 are truly unique. However, attempts to assess the true prevalence of phage superspreaders via metagenomics would likely be confounded by a number of factors. The accumulation of viral genome sequences has rapidly outpaced their functional annotation (37), and the vast number of hypothetical phage proteins with unknown functions greatly complicates efforts to quantify the absence of a particular phenotype (e.g., plasmid degradation) across sequence space. Moreover, our observation that λ*c*I^−^ also promotes efficient plasmid transformation suggests that an assessment of SUSP-specific marker genes, a common approach for evaluating viral diversity (38), would not accurately quantify the breadth of the superspreading phenomenon. In other words, even if SUSP-like gene signatures were found to be uncommon in metagenomic data sets, it would not necessarily follow that SUSP-like phenotypes are similarly uncommon in natural environments. Indeed, we note that T4-like cyanophages, the oceans’ single most abundant group of viruses, differ from T4—but resemble SUSP1 and SUSP2—in lacking homologs of *denA*, *denB*, and *ndd* (39). If the superspreading phenotype is indeed associated with the absence of plasmid-degrading gene products, phage superspreaders could conceivably hail from diverse viral lineages and occur in diverse ecological settings. More importantly, and regardless of their specific mechanism(s), we propose that phage superspreaders may act as key drivers of horizontal gene transfer and bacterial evolution in natural environments.

Our findings also have important implications for phage therapy, which has experienced a resurgence in recent years expressly because of its potential to combat antibiotic-resistant infections (1). Since much of this resistance is plasmid borne (4, 5), the dispersal of intact plasmid DNA during phage therapy would be clinically undesirable, especially since certain classes of antibiotics increase the competence of certain bacterial pathogens (40, 41). Although our results do not, in our opinion, diminish the broader utility of phage therapy, we recommend that therapeutic phage candidates be prescreened to prevent the inadvertent use of superspreader phages in biomedical applications.

## MATERIALS AND METHODS

For a complete description of the materials and methods used in this study, see Text S1 in the supplemental material.

### Bacteriophage isolation and validation.

All of the bacteria and bacteriophages used in this study were grown at 37°C. Phages were isolated via direct plating from environmental samples (soil, feces, canal water, or stream water) collected in the metropolitan areas of Miami, FL, and Washington, DC. Wild-type *E. coli* MG1655 was used as the host strain. Phages that formed clear plaques, but not those that formed turbid plaques and thus presumed to be lysogenic, were retained for further characterization. Specifically, high-titer phage lysates (>10^9^ PFU/ml) were propagated in LB^+^ (LB supplemented with 10 mM CaCl_2_ and 10 mM MgCl_2_) from single plaques with wild-type *E. coli* MG1655 as the host strain, centrifuged to remove bulk debris, and then purified via polyethylene glycol (PEG) precipitation. DNA was isolated from PEG-purified phage lysates via phenol-chloroform extraction and precipitated with pure isopropanol and NaCl. The resulting nucleic acid pellets were rinsed with 70% ethanol, air dried, and resuspended in ultrapure water. Phage DNA samples were digested separately with HindIII and XbaI and visualized via gel electrophoresis, thereby allowing the compilation of a collection of genetically distinct phages. In total, 13 distinct clear-plaque-forming phage isolates were obtained from various locations in Miami-Dade County, FL, and seven such phages were isolated from various sites in Montgomery County, MD.

### Lysis-and-transformation assays.

High-titer (>10^9^ PFU/ml) phage stock lysates were propagated from single plaques as described above, passed through 0.44-µm filters to remove residual bacteria and cellular debris, and stored at 4°C until use. In subsequent lysis-and-transformation assays, 1-ml aliquots of stock phage lysates were supplemented with 100 µl of 5× LB^+^, inoculated with 10 µl of an overnight culture of *E. coli* MG1655 bearing plasmid pBAD24 (here, MG/pBAD24), and then incubated with aeration for 2 h. In these assays, phages outnumbered bacteria in each reaction tube by at least 10-fold, thereby ensuring equivalent killing of bacteria despite differences in phage replication kinetics. Following incubation, the resulting “secondary” phage lysates were transferred to microcentrifuge tubes and thrice centrifuged to remove residual bacteria and bulk debris. DNA was purified from these bacterium-free supernatants via phenol-chloroform extraction as described above, resuspended in ultrapure water, and immediately used in transformation reactions. Competent *E. coli* MG1655 cells were generated via the transformation-and-storage solution (TSS) method (14), mixed with 1/6 volume of extracted DNA, and incubated on ice for 90 min. Thereafter, cell mixtures were resuspended in 1 ml of SOC medium (2% tryptone w/v, 0.5% yeast extract w/v, 10 mM NaCl, 2.5 mM KCl, 10 mM MgCl_2_, 20 mM glucose) and incubated for 1 h with aeration. Appropriate culture volumes were plated on LB plates containing 50 µg/ml ampicillin and incubated overnight. Ampicillin-resistant transformants were subsequently enumerated and multiplied by appropriate dilution factors to quantify the total number of transformants resulting from each phage lysate (Fig. 1).

Plasmids pSP102m5, pOAR31, and pπγ were transformed into wild-type *E. coli* MG1655 (see Table S2). Lysis-and-transformation assays involving those strains (Fig. 2) were conducted exactly as described for MG/pBAD24 above, except that selective plates and media contained 50 µg/ml kanamycin (MG/pOAR31 and MG/pπγ) or 25 µg/ml chloramphenicol (MG/pSP102m5) instead of ampicillin. As a positive control, plasmid DNA was extracted from an equal volume (10 µl) of overnight culture of each strain (QIAprep Miniprep kit; Qiagen) in accordance with the manufacturer’s instructions and transformed exactly as described above.

### Transduction and MOI assays.

To assess the relative importance of phage packaging of bacterial DNA (transduction) in the above-mentioned assays (see Fig. S1), an additional set of SUSP2 lysates of MG/pBAD24 were centrifuged for 1 h at 21,000 × *g*. DNA was extracted from the resulting supernatants and transformed into TSS-competent *E. coli* MG1655 cells exactly as described above. Phage titers before and after centrifugation were quantified via plaque assay. To assess the importance of the MOI in our assays (see Fig. S2), additional lysis-and-transformation assays were established in which MG/pBAD24 cells were infected with 100-fold fewer SUSP2 phage particles than usual (starting MOI of <1 instead of >10). DNA was extracted from the supernatants of these lysates and transformed into TSS-competent *E. coli* MG1655 cells exactly as described above.

### Additional lysis-and-transformation assays.

Additional lysis-and-transformation assays (Fig. 3) were conducted to confirm that the activity of SUSP1 and SUSP2 is not restricted to a specific plasmid donor strain, plasmid recipient strain, or transformation protocol. Specifically, this second set of assays utilized *E. coli* Le392 (instead of *E. coli* MG1655) bearing plasmid pBAD24 as the plasmid donor strain, wild-type *E. coli* DH5α (instead of *E. coli* MG1655) as the plasmid recipient strain, and a transformation protocol involving calcium chloride and heat shock (instead of TSS).

High-titer stock lysates of phages SUSP2, wild-type T4, T4GT7, and λ*c*I^−^ (see Table S2) were prepared in a wild-type *E. coli* Le392 background. Meanwhile, plasmid pBAD24 was extracted from MG/pBAD24 (QIAprep Miniprep kit; Qiagen) and transformed into wild-type *E. coli* Le392 to create Le/pBAD24.

Lysis-and-transformation reaction mixtures, each consisting of 1 ml of bacterium-free phage lysate (SUSP2, T4, T4GT7, or λ*cI*^*−*^), 100 µl of 5× LB^+^, and 10 µl of an overnight Le/pBAD24 culture, were incubated with aeration for 2 h. DNA was extracted from these secondary lysates with phenol-chloroform exactly as described above, except that the resulting nucleic acid pellets were resuspended in buffer EB (10 mM Tris-Cl, pH 8.5) rather than ultrapure water. Plasmid DNA was also extracted from 10-µl aliquots of an overnight Le/pBAD24 culture (QIAprep Miniprep kit; Qiagen) in accordance with the manufacturer’s instructions, eluted with buffer EB, and retained as a positive control for transformation. Thereafter, competent *E. coli* DH5α cells were mixed with 1/9 volume of extracted DNA in prechilled PCR tubes. Following the completion of a heat shock thermocycling program, the newly transformed cells were incubated in 1 ml of SOC medium at 37°C with aeration for 90 min. Appropriate culture volumes were plated on selective media and incubated overnight. Ampicillin-resistant transformants were subsequently enumerated and multiplied by appropriate dilution factors to quantify the total number of transformants resulting from each phage lysate.

### ddPCR.

ddPCR (Bio-Rad) was conducted to quantify the levels of intact plasmid and chromosomal DNA in phage lysates (see Fig. S3). PCR primers were designed to amplify intact 130-bp fragments from sequenced plasmid pBAD24 and from the *rpoS* gene of *E. coli* MG1655 (see Table S2). Briefly, DNA was extracted from bacterium-free supernatants derived from MG/pBAD24 lysis-and-transformation assays as described above, resuspended in ultrapure water, and serially diluted. Extraction and purification of plasmid DNA via a commercial kit (QIAprep Miniprep kit; Qiagen) from an equal volume (10 µl) of a phage-free overnight bacterial culture served as the positive control for plasmid amplification. Template-free negative controls in which additional ultrapure water replaced DNA confirmed the absence of meaningful contamination. All ddPCR procedures, including pre-PCR droplet generation and post-PCR sample reading, were conducted in accordance with the manufacturer’s instructions. ddPCR data were analyzed with the QuantaSoft software program, version 1.7.4.0917 (Bio-Rad).

### Long-range PCR and TapeStation analysis.

Long-range Phusion PCR (Thermo Scientific) was combined with the TapeStation platform (Agilent Technologies) to amplify and quantify intact plasmid pBAD24 extracted from phage lysates (see Fig. S4). In contrast to the Southern blot assay and similar techniques, TapeStation offers automated sample processing and accurate digital quantification. PCR primers were designed to amplify nearly the entire pBAD24 plasmid (4,480 of 4,542 bp or 98.6%; see Table S2). Subsequently, DNA was extracted from bacterium-free phage lysates arising from lysis-and-transformation assays of MG/pBAD24 as described above, resuspended in ultrapure water, and further diluted 10-fold. Plasmid DNA was extracted from 10 µl of an overnight MG/pBAD24 culture via a commercial kit (QIAprep Miniprep kit; Qiagen), also diluted 10-fold, and used as the positive control. Following PCR completion, samples were diluted 1:10 in D5000 ScreenTape buffer and assayed via D5000 ScreenTape.

### Transmission election microscopy.

One-milliliter aliquots of high-titer, bacterium-free SUSP1 and SUSP2 stock lysates were centrifuged for 60 min at 21,000 × *g*. The supernatants were aspirated, and the resulting phage pellets were resuspended in deionized water. Phage drops were deposited on 0.25% Formvar-coated copper grids, allowed to adsorb for 1 min, stained with 2% methanolic uranyl acetate for 15 s, rinsed with 1 drop of deionized water, and gently blotted dry with filter paper. Grids were visualized with a JEOL 1400 microscope at 80 kV and imaged with a Gatan digital camera. Images were minimally enhanced for brightness and contrast (Adobe Photoshop) but not otherwise manipulated (see Fig. S6).

### Phage genome sequencing and analysis.

DNA was extracted from high-titer, PEG-purified phage lysates with phenol-chloroform exactly as described above and sequenced by using paired-end reads (Nextera XT kit and MiSeq; Illumina). Sequencing data were trimmed, filtered, and assembled in CLC Genomics workbench 8.0.1 to yield contigs of 90.76 kb (SUSP1) and 88.70 kb (SUSP2) with average coverages of 9,983× and 7,790×, respectively. These putative sequences were validated by Phusion PCR (Thermo Scientific) tiling at 16-kb intervals with primers predicted from the draft genome sequences (see Table S2). The sequences were further verified by targeted Sanger sequencing of low-coverage regions (BigDye Terminator v1.1; Life Technologies, Inc.) and alignment with Sequencher v 5.2 (Gene Codes Corporation). Sanger sequencing of putative ends revealed a circularly permuted genome. Putative open reading frames were identified with GeneMarkS (42) and further validated with RAST (43). Putative tRNA genes were identified with tRNAscan-SE (44). Genome maps of SUSP1 and SUSP2 (see Fig. S5) were constructed with DNAPlotter (45).

### Natural transformation assays.

Lysis-and-transformation assays involving MG/pπγ (*E. coli* MG1655 bearing kanamycin resistance plasmid pπγ) and phages SUSP2 and T4 were conducted exactly as described above (i.e., incubating 1 ml of stock phage lysate, 100 µl of 5× LB^+^, and 10 µl of an overnight MG/pπγ culture for 2 h at 37°C with aeration). The resulting secondary lysates were thrice centrifuged at 14,000 × *g* to remove any surviving bacteria (as confirmed by plating on selective media and by overnight incubation) but not otherwise manipulated.

Soil samples were sterilely collected from multiple locations in Montgomery County, MD, and Laramie County, WY. Bacteria were isolated from these samples by simple rinsing with TMG (Tris-MgSO_4_-gelatin) buffer. Immediately thereafter, bacterium-free SUSP2 lysate, T4 lysate, or LB medium was inoculated with 100 µl of resuspended soil bacteria and incubated overnight at 37°C with aeration. Following incubation, samples were plated on media containing 50 µg/ml kanamycin to quantify the number of kanamycin-resistant bacteria arising from exposure to superspreading phage, nonsuperspreading phage, and control media, respectively (Fig. 4). Samples were also plated on nonselective media to confirm that there were no significant differences in the total number of bacteria, irrespective of kanamycin resistance, in each overnight culture.

### 16S rRNA sequencing.

A bacterial strain abundant in Wyoming soil samples (WY10) was established in pure culture and grown overnight. Bacterial cells were lysed with SDS, and DNA was isolated via phenol-chloroform extraction. PCRs were conducted with two universal 16S rRNA primer pairs, 27F/584R and 928F/1492R (see Table S2). Each resulting PCR product was purified and Sanger sequenced separately with its forward and reverse primers. Sequence chromatograms were visually inspected in Sequencher 5.4.5 (Gene Codes Corporation) and queried against existing sequence databases (NCBI BLASTn) to identify the genus of WY10. On the basis of significant (≥99%) homology to >50 individual *Bacillus* sp. genome sequences, WY10 was assigned to that same genus. To further establish the identity of WY10, extracted genomic DNA was also sequenced via next-generation sequencing (Nextera XT kit and MiSeq; Illumina). Sequencing data were trimmed, filtered, and assembled in CLC Genomics workbench 8.0.1, and the largest resulting contig (461.7 kb) was queried against existing sequence databases (NCBI BLASTn).

### Coculture experiments.

WY10 was determined to be sensitive to kanamycin and resistant to phages SUSP2 and T4 via plating on selective media and via plaque assay, respectively. Four-milliliter aliquots of LB medium were inoculated with 10^8^ WY10 cells, 10^8^ MG/pπγ cells, and 10^7^ phage plaque-forming units of either SUSP2 or T4 and then incubated overnight at 37°C with aeration. Phage-free controls containing the same bacterial inocula and additional controls in which 10^8^ wild-type *E. coli* MG1655 cells were substituted for MG/pπγ were identically established. Following overnight incubation, samples were serially diluted and plated on selective and nonselective media to establish the number of kanamycin-resistant WY10 cells arising from each experimental microcosm (Fig. 5). Importantly, the colony morphotype of WY10 is readily distinguishable from that of *E. coli*, thereby allowing their visual differentiation upon plating. Plasmid DNA was extracted from randomly selected kanamycin-resistant WY10 colonies as described above (QIAprep Miniprep kit; Qiagen) and assayed via Genomic ScreenTape (TapeStation platform; Agilent Technologies) to confirm the presence of plasmid pπγ (see Fig. S7).

### Accession number(s).

The complete genome sequences of phages SUSP1 and SUSP2 have been deposited in GenBank and assigned accession numbers KT454805 and KT454806, respectively.
