# Supplementary material for: Novel “Superspreader” Bacteriophages Promote Horizontal Gene Transfer by Transformation
Source: mBio. 2017 Jan 17;8(1):e02115-16. doi: 10.1128/mBio.02115-16 (PMC5241400; doi:10.1128/mBio.02115-16)
Supplement: TEXT S1 [file mbo002173145s1.docx]

**Supplementary Materials and Methods**

**Bacteriophage isolation**

All bacteria and bacteriophages were grown at 37°C. Phages were isolated via direct plating from environmental samples collected in the metropolitan areas of Miami, FL, and Washington, D.C.  Samples of soil or feces were added to 1 ml of sterile 0.9% saline, vortexed, and centrifuged for 2 minutes at 14,000 x *g*.  The resulting supernatants were mixed with 100 µl *Escherichia coli* MG1655 overnight culture and 3 ml of 0.7% top agar, plated as overlays on LB plates (1% tryptone, 1% NaCl, 0.5% yeast extract wt/v), and incubated overnight.  Samples of stream or canal water were mixed directly with bacteria and top agar, poured on LB plates, and incubated overnight. Phages which formed clear plaques, but not those which formed turbid plaques and therefore presumed to be lysogenic, were retained for further characterization and twice re-plated from single plaques to ensure purity. In total, 13 distinct clear plaque-forming phage isolates were obtained from various locations in Miami-Dade County, FL, and seven such phages were isolated from various sites in Montgomery County, MD.

**Bacteriophage genome extraction and digestion**

High-titer phage lysates (>10^9^ pfu/ml) were propagated in LB+ (LB supplemented with 10 mM CaCl_2_ and 10 mM MgCl_2_) from single plaques with wild-type *E. coli* MG1655 as the host strain. Following complete clearing, lysates were centrifuged for 4 minutes at 14,000 x *g* and purified via PEG precipitation. Briefly, 10 ml phage lysate aliquots were mixed with 2.5 ml of PEG solution (20% PEG 8000 wt/v; 2.5 M NaCl) and incubated on ice for one hour. Thereafter, the samples were centrifuged for 60 minutes at 4,500 x *g*, the supernatants aspirated, and the phage pellets re-suspended in 1 ml STE buffer (10 mM Tris, 1 mM EDTA, 100 mM NaCl). DNA was extracted from these PEG-purified phage lysates via the addition of 220 µl phenol:chloroform:isoamyl alcohol (25:24:1, Sigma), centrifugation for 4 minutes at 14,000 x *g*, extraction of the uppermost 750 µl (aqueous phase), and precipitation with 650 µl pure isopropanol and 75 µl 5 M NaCl. The resulting nucleic acid pellets were rinsed with 70% ethanol, dried briefly at 37°C, and re-suspended in 100 µl ultrapure water. Each DNA sample was digested separately with the restriction enzymes HindIII and XbaI for one hour at 37°C and run on a 1% agarose gel. Two different DNA banding patterns were thus obtained for each phage isolate, allowing the compilation of a library of genomically distinct phages.

**Lysis-and-transformation assays**

High-titer (>10^9^ pfu/ml) phage stock (“primary”) lysates were propagated from single plaques as described above, passed through 0.44 µm filters to remove intact bacteria and cellular debris, and stored at 4°C until use. *E. coli* MG1655 bearing the ampicillin-resistance plasmid pBAD24 (MG/pBAD24) was used as the plasmid donor strain in these experiments. 1 ml aliquots from these stock phage lysates were supplemented with 100 µl 5x LB+ and inoculated with 10 µl *E. coli* MG/pBAD24 overnight culture (<10^10^ CFU/ml; each culture titered in duplicate with use for subsequent “per cell” data normalization), then incubated at 37°C with aeration for 2 hours to generate fresh “secondary” phage lysates. Upon inoculation of these secondary lysates, phages outnumbered bacteria in each tube by at least 10-fold, thereby ensuring equal bacterial killing despite differences in phage replication kinetics.

Following the two-hour infection period, freshly prepared secondary phage lysates were transferred to microfuge tubes and thrice-centrifuged for 2 minutes at 14,000 x *g* and repeatedly aspirated to remove intact bacteria and cellular debris. These secondary lysate supernatants were determined to be free of bacteria via plating on selective media (no colony formation) and via overnight incubation at 37°C (no resulting turbidity). DNA was extracted from 900 µl aliquots of these bacteria-free supernatants via phenol-chloroform extraction as described above, re-suspended in 100 µl ultrapure water, and used in transformation reactions less than 30 minutes later.

Competent cells were generated as described previously **[14]**, with some modifications. Briefly, *E. coli* MG1655 overnight culture was diluted 1:50 in fresh LB medium, grown with aeration at 37°C to OD_600_ 0.60-0.65, centrifuged at 4°C for 5 minutes at 4,500 x *g*, and re-suspended in 1/10^th^ volume ice-cold TSS (10% PEG 8000 wt/v, 5% DMSO, 30 mM MgCl_2_). 120 µl aliquots of competent cells were gently mixed with 20 µl extracted DNA and incubated on ice for 90 minutes. Thereafter, each tube was supplemented with 1 ml SOC medium (2% tryptone wt/v, 0.5% yeast extract wt/v, 10 mM NaCl, 2.5 mM KCl, 10 mM MgCl_2_, 20 mM glucose) and incubated for 1 hour at 37°C with aeration. Appropriate culture volumes were plated on LB plates containing 50 µg/ml ampicillin (“LB + Amp plates”) and incubated overnight. Ampicillin-resistant transformants on each plate were enumerated the following morning and multiplied by relevant dilution factors (a constant 5x dilution, since only 20 µl of the 100 µl DNA re-suspension volume was used per reaction, plus additional dilutions to compensate for the plating of different cell volumes on LB + Amp plates) to quantify the total number of transformants resulting from each phage lysate.

Plasmids pSP102m5 (mini-P1 origin with copy-up mutation), pOAR31 (RSF1010 origin), and pπγ (R6K origin) were kindly provided by D. Chattoraj (NCI) and transformed into wild-type *E. coli* MG1655 as described above (**Table S2**). Lysis-and-transformation assays involving those strains were conducted exactly as described for MG/pBAD24 above, except that relevant plates and media contained 50 µg/ml kanamycin (MG/pOAR31 and MG/pπγ) or 25 µg/ml chloramphenicol (MG/pSP102m5) instead of ampicillin. As a positive control, plasmid DNA was extracted from 10 µl aliquots of overnight cultures from all four strains (QiaPrep Miniprep kit, Qiagen) as per the manufacturer’s instructions and transformed exactly as described above.

**Transduction and MOI assays**

To assess the relative importance of phage DNA packaging (transduction) in the above-mentioned assays, an additional set of SUSP2 lysates of MG/pBAD24 were centrifuged for 1 hour at 21,000 x *g*. DNA was extracted from the resulting supernatants and transformed into TSS-competent *E. coli* MG1655 exactly as described above. Phage titers before and after centrifugation were quantified via plaque assay (*i.e.*, 0.7% agar overlays on LB plates). To assess the importance of multiplicity of infection (MOI) in our assays (**Fig. S2**), additional lysis-and-transformation assays, each containing 10 µl MG/pBAD24 overnight culture, 10 µl of high-titer SUSP2 lysate, and 1 ml LB+ (final MOI <1), were established. Following the customary two-hour incubation period, DNA was extracted from the supernatants of these lysates and transformed into TSS-competent *E. coli* MG1655 cells exactly as described above.

**Additional lysis-and-transformation assays**

Additional lysis-and-transformation assays (**Fig. 3**) were conducted via a different procedure to confirm that our results were not attributable to a specific plasmid donor strain, plasmid recipient strain, or transformation protocol. Specifically, this second set of assays utilized *E. coli* Le392 (instead of *E. coli* MG1655), bearing the plasmid pBAD24, as the plasmid donor strain; wild-type *E. coli* DH5α (instead of *E. coli* MG1655) as the plasmid recipient strain, and a transformation protocol involving calcium chloride and heat shock rather than re-suspension of cells and DNA in TSS. Competent cells were prepared by growing 500 ml of *E. coli* DH5α culture to OD_600_ 0.5-0.6 in a 2000 ml flask, which was then briefly swirled in ice water and incubated on ice for 30 minutes. Chilled cells were removed in 40 ml increments to Falcon tubes and centrifuged for 10 minutes at 4°C and 5000 revolutions per minute, washed twice with ice-cold water, and re-suspended in 25 ml ice-cold 100 mM CaCl_2_ solution. These cells were then incubated on ice for an additional 30 minutes, spun down at 4°C, and re-suspended in 4 ml of 50 mM CaCl_2_/15% glycerol solution. 250 µl cell aliquots were added to pre-chilled microfuge tubes and frozen at -80°C until use.

High-titer stock lysates of phages SUSP2, wild-type T4, T4GT7 (partial endonuclease deletion mutant of T4), and λ*cI^-^* (lytic mutant of λ) (**Table S2**) were prepared in a wild-type *E. coli* Le392 background. Phage T4GT7 was kindly provided by N. Majdalani (NCI). Meanwhile, plasmid pBAD24 was extracted from MG/pBAD24 (QiaPrep Miniprep Kit, Qiagen) and transformed into wild-type *E. coli* Le392 to create Le/pBAD24.

Reaction tubes containing 1 ml of bacteria-free phage lysate (SUSP2, T4, T4GT7, or λ*cI^-^*), 100 µl 5x LB+, and 10 µl overnight Le/pBAD24 culture were incubated with aeration for two hours at 37°C. DNA was extracted from these secondary lysates with phenol-chloroform exactly as described above, except that the resulting nucleic acid pellets were re-suspended in 100 µl Buffer EB (10 mM Tris-Cl, pH 8.5) instead of 100 µl ultrapure water. Plasmid DNA was also extracted from 10 µl aliquots of overnight Le/pBAD24 culture (QiaPrep Miniprep kit, Qiagen) as per the manufacturer’s instructions, eluted with 100 µl Buffer EB, and retained as a positive control for transformation. Thereafter, 5 µl aliquots of extracted DNA were mixed with 45 µl aliquots of competent *E. coli* DH5α cells, which had been thawed on ice, in pre-chilled PCR tubes. Following the completion of a heat shock thermocycling program (30 minutes at 4°C, one minute at 42°C, and two additional minutes at 4°C), the newly transformed cells were removed to microfuge tubes containing 1 ml SOC medium and incubated at 37°C with aeration for 90 minutes. Appropriate culture volumes were plated on LB + Amp plates and incubated overnight. Ampicillin-resistant colonies were enumerated the following morning and multiplied by relevant dilution factors as described above to quantify the total number of transformants resulting from each phage lysate.

**Digital droplet PCR**

Digital droplet PCR (ddPCR; BioRad) was conducted to quantify the levels of intact plasmid and chromosomal DNA in phage lysates. PCR primers were designed to amplify intact 130-bp fragments from the sequenced plasmid pBAD24 and from the *rpoS* gene of *E. coli* MG1655 (**Table S2**). Briefly, DNA was extracted from bacteria-free supernatants derived from MG/pBAD24 lysis-and-transformation assays as described above, re-suspended in 100 µl ultrapure water, and serially diluted 10000-fold to yield approximately 1000 copies per well upon amplification. Extraction and purification of plasmid DNA via a commercial kit (QiaPrep Miniprep kit, Qiagen) from an equal volume (10 µl) of overnight bacterial culture unexposed to phage served as the positive control for plasmid amplification. ddPCR reaction mixtures consisted of 2.2 µl diluted template DNA, 8 µl ultrapure water, 0.4 µl of both forward and reverse primers (180 nM working concentration of each), and 11 µl QX200 2x EvaGreen Supermix (BioRad). Template-free negative controls, in which an extra 2.2 µl of ultrapure water was used instead of re-suspended DNA, confirmed the absence of significant contamination. The following PCR program was used: 5 minutes at 95°C, 40 cycles of [30 seconds at 95°C and 1 minute at 60°C], 5 minutes at 4°C, and 5 minutes at 90°C. All other ddPCR procedures, including pre-PCR droplet generation and post-PCR sample reading, were conducted as per the manufacturer’s instructions. All ddPCR data were analyzed with the QuantaSoft software program, version 1.7.4.0917 (BioRad).

**Long-range PCR and TapeStation Analysis**

Because the 4542-bp pBAD24 plasmid is too large to amplify completely via either ddPCR or conventional quantitative PCR, long-range Phusion PCR (Thermo Scientific) was combined with the TapeStation platform (Agilent Technologies) to amplify and quantify intact plasmid pBAD24 extracted from phage lysates. In contrast to the Southern blot and other semi-quantitative techniques for DNA analysis, TapeStation offers automated sample processing and accurate digital quantification, and the platform is widely used to measure exact DNA concentrations prior to library preparation for next-generation sequencing. PCR primers (**Table S2**) were designed to amplify nearly the entire pBAD24 plasmid (4480 of 4542 bp, or 98.6%). Subsequently, DNA was extracted from bacteria-free phage lysates arising from lysis-and-transformation assays of MG/pBAD24 as described above, re-suspended in 100 µl ultrapure water, and further diluted tenfold. Extraction and purification of plasmid DNA via a commercial kit (QiaPrep Miniprep kit, Qiagen) from 10 µl overnight MG/pBAD24 culture was also diluted tenfold and used as the positive control. PCR reaction mixtures consisted of 5 µl diluted template DNA, 4 µl ultrapure water, 0.5 µl of both forward and reverse primers (225 nM working concentration of each), and 10 µl 2x Phusion Mastermix (Thermo Scientific). The following PCR program was used: 30 seconds at 98°C, 22 cycles of [10 seconds at 98°C, 20 seconds at 60°C, 5 minutes at 72°C], and 10 minutes at 72°C. Following Phusion PCR completion, 1 µl of each reaction mixture was added to 10 µl D5000 ScreenTape buffer, vortexed for 30 seconds, centrifuged briefly, and assayed via D5000 ScreenTape. Since equal volumes of extracted DNA (which, in turn, contained the byproducts of equal amounts of bacterial killing) were used as template, the intensity of Tape band peaks was presumed to correlate with the extent of intact pBAD24 plasmid survival in various phage lysates.

**Transmission election microscopy**

1 ml aliquots of high-titer, bacteria-free SUSP1 and SUSP2 stock lysates were centrifuged for 60 minutes at 21,000 x *g*. The supernatants were aspirated and the resulting phage pellets re-suspended in 50 µl deionized water.  3 µl drops of re-suspended phage particles were deposited on 0.25% Formvar-coated copper grids, allowed to absorb for one minute, stained with 2 µl of 2% methanolic uranyl acetate for 15 seconds, rinsed with one drop of deionized water, and gently blotted dry with filter paper.  Grids were visualized with a Jeol 1400 microscope at 80 kV and imaged with a Gatan digital camera.  Images were minimally enhanced for brightness and contrast (Adobe Photoshop) but not otherwise manipulated.

**Phage genome sequencing and analysis**

DNA was extracted from high-titer, purified phage lysates with phenol-chloroform exactly as described above and sequenced using paired-end reads (Nextera XT Kit and MiSeq, Illumina). Sequencing data were trimmed, filtered, and assembled in CLC Genomics Workbench 8.0.1 to yield contigs of 90.76 kb (SUSP1) and 88.70 kb (SUSP2) with average coverage of 9,983x and 7,790x, respectively. These putative sequences were validated by Phusion PCR (Thermo Scientific) tiling at 16 kb intervals with primers predicted from the draft genome sequence (**Table S2**). The sequences were further verified by targeted Sanger sequencing of low-coverage regions (BigDye Terminator v1.1, Life Technologies) and alignment with Sequencher v5.2 (Gene Codes Corporation). Sanger sequencing of putative ends revealed a circularly permuted genome. Putative open reading frames were identified with GeneMarkS **[42]** and further validated with RAST **[43]**. Putative tRNA genes were identified with tRNAscan-SE **[44]**. Genome maps of SUSP1 and SUSP2 were constructed with DNAPlotter **[45]**.

The complete genome sequences of phages SUSP1 and SUSP2 have been deposited in GenBank with accession numbers **KT454805** and **KT454806**, respectively.

**Natural transformation assays**

Lysis-and-transformation assays involving MG/pπγ *(E. coli* MG1655 bearing the kanamycin resistance plasmid πγ) and phages SUSP2 and T4 were conducted exactly as described above (*i.e.,* incubating 1 ml stock phage lysate, 100 µl 5x LB+, and 10 µl overnight MG/pπγ culture for two hours at 37°C with aeration). The resulting secondary lysates were centrifuged three times at 14,000 x *g* to remove any surviving bacteria. Successful removal of bacteria was confirmed by plating on media containing 50 µg/ml kanamycin (“LB + Kan plates”; no growth) and by overnight incubation in LB media (no turbidity).

Soil samples were sterilely collected from three geographically discrete locations in Montgomery County, MD, and two discrete locations in Laramie Country, WY, and kept at 4°C until use. Immediately following the preparation of SUSP2 and T4 secondary lysates of MG/pπγ, 3 grams of individual soil samples were mixed with 3 ml TMG (Tris-MgSO_4_-gelatin buffer, pH 7.5), vortexed for 1 minute, and centrifuged at 2,000 x *g* for 5 minutes. The uppermost 1.5 ml of the supernatants were removed to sterile microfuge tubes and centrifuged again for 5 minutes at 2,000 x *g*. The uppermost 1 ml fractions of these second supernatants contained no visible soil particles and were retained as unbiased samples of soil bacteria.

Immediately thereafter, 900 µl aliquots of bacteria-free SUSP2 lysates, bacteria-free T4 lysates, or LB medium were inoculated with 100 µl of re-suspended soil bacteria and incubated overnight at 37°C with aeration. Incubated samples were retained at 4°C for approximately 8 hours, serially diluted as appropriate, and plated on non-selective LB plates (to determine the number of total CFUs) and LB + Kan plates (to determine the number of kanamycin-resistant CFUs), thereby quantifying the degree of antibiotic resistance in samples of environmental bacteria following exposure to superspreading phage, non-superspreading phage, and control medium, respectively.

**16S rRNA sequencing**

A particular bacterial colony morphotype was abundant on LB + Kan plates following the growth of Wyoming soil bacteria in SUSP2 lysates of MG/pπγ. Additional soil samples from the same site were rinsed with TMG and plated on LB plates, whereupon the same colony morphology was again observed. This bacterial isolate (“WY10”) was established in pure culture and propagated overnight from a single colony in 5 ml LB medium. 1 ml bacterial culture was mixed with 200 µl of 10% sodium dodecyl sulfate (SDS) and 100 µl of 0.5 M EDTA, then incubated at 37°C with aeration for 20 minutes. Thereafter, DNA was extracted via the addition of 220 µl phenol:chloroform:isoamyl alcohol (25:24:1, Sigma), centrifugation for 4 minutes at 14,000 x *g*, extraction of the uppermost 750 µl (aqueous phase), and precipitation with 650 µl pure isopropanol and 75 µl 5M NaCl. The resulting nucleic acid pellets were rinsed with 70% ethanol, dried briefly at 37°C, and re-suspended in 30 µl of ultrapure water.

PCR reactions were carried out with PuReTaq Ready-to-Go PCR Beads (GE Healthcare). Reaction mixtures consisted of 15 µl ultrapure water, 3 µl of extracted WY10 DNA, and 1 µl each of forward and reverse primer (**Table S2**). The following PCR program was used: 5 minutes at 95°C, 30 cycles of [30 seconds at 95°C, 30 seconds at 60°C, and 1 minute at 72°C], and 10 minutes at 72°C. PCR products were purified with Peforma gel filtration cartridges (EdgeBio) and used as templates for Sanger sequencing reactions. Sanger sequencing reaction mixtures consisted of 6 µl ultrapure water, 7 µl PCR product, 1 µl of either forward or reverse primer (the same as were used to generate that PCR product), 2 µl BigDye Terminator dye v.1.1, and 4 µl of 2.5x BigDye sequencing buffer. The following amplification program was used: 30 cycles of [10 seconds at 96° C, 5 seconds at 50° C, and 4 minutes at 60° C]. The resulting products were purified with Peforma gel filtration cartridges (EdgeBio) and dried by vacufuge. Dehydrated PCR products were sequenced with a 3130xl Genetic Analyzer (Applied Biosystems) as per the manufacturer’s instructions. Chromatograms were visualized and verified in Sequencher 5.4.5 (Gene Codes Corporation), and the resulting sequences were queried against the NCBI genome database (blastn megablast) to establish the genus of WY10. Based on significant (≥99%) homology to >50 individual *Bacillus* sp. genome sequences, WY10 was assigned to that same genus.

To further establish the identity of WY10, extracted genomic DNA was also sequenced via next-generation sequencing (Nextera XT Kit and MiSeq, Illumina). Sequencing data were trimmed, filtered, and assembled in CLC Genomics Workbench 8.0.1, and the largest contig (461.7 kb) was queried against existing sequence databases (NCBI blastn).

**Co-culture experiments**

Overnight cultures of WY10, wild-type *E. coli* MG1655, and MG/pπγ were propagated from single colonies, serially diluted, and titered on LB plates. High-titer, bacteria-free lysates of phages SUSP2 and T4 were prepared from single plaques and titered via plaque assay. WY10 was determined to be kanamycin-sensitive and SUSP2- and T4-resistant via plating on selective media and via plaque assay, respectively. Subsequently, 4 ml aliquots of LB medium were inoculated with 10^8^ WY10 cells, 10^8^ MG/pπγ cells, and 10^7^ phage virions (either SUSP2 or T4), then incubated overnight at 37°C with aeration. Phage-free controls containing the same bacterial inocula, and additional controls in which 10^8^ wild-type *E. coli* MG1655 cells were used in place of MG/pπγ, were also established in the same manner. Following overnight incubation, samples were serially diluted and plated on LB plates and LB + Kan plates to establish the proportion of kanamycin-resistant WY10 cells in each experimental microcosm. Because each microcosm contained not just WY10 but also SUSP2- or T4-resistant *E. coli* which had propagated overnight, plates were pre-treated with 10^8^ T7 phage prior to bacterial plating. Pre-treatment with T7 almost completely eliminated these pre-existing phage-resistant *E. coli* (but not WY10, which was not infectable by T7) and enabled the plating of less dilute samples, thus increasing experimental accuracy. Importantly, the colony morphotype of WY10 was readily distinguishable from that of *E. coli*, allowing the differentiation of WY10 and *E. coli* upon plating.

To confirm the assumption that kanamycin-resistant WY10 colonies contained plasmid pπγ, several such colonies were individually picked and propagated overnight in 5 ml LB medium. Thereafter, plasmid DNA was isolated from 800 µl overnight culture (QiaPrep Miniprep kit, Qiagen) and from equal overnight culture volumes of wild-type WY10 (negative control) and MG/pπγ (positive control). 1 µl Miniprep product aliquots were re-suspended in 10 µl Genomic ScreenTape buffer and run on the TapeStation platform (Agilent Technologies) to visualize plasmid bands.
