# Supplementary figures and images for: Novel “Superspreader” Bacteriophages Promote Horizontal Gene Transfer by Transformation
Source: mBio. 2017 Jan 17;8(1):e02115-16. doi: 10.1128/mBio.02115-16 (PMC5241400; doi:10.1128/mBio.02115-16)

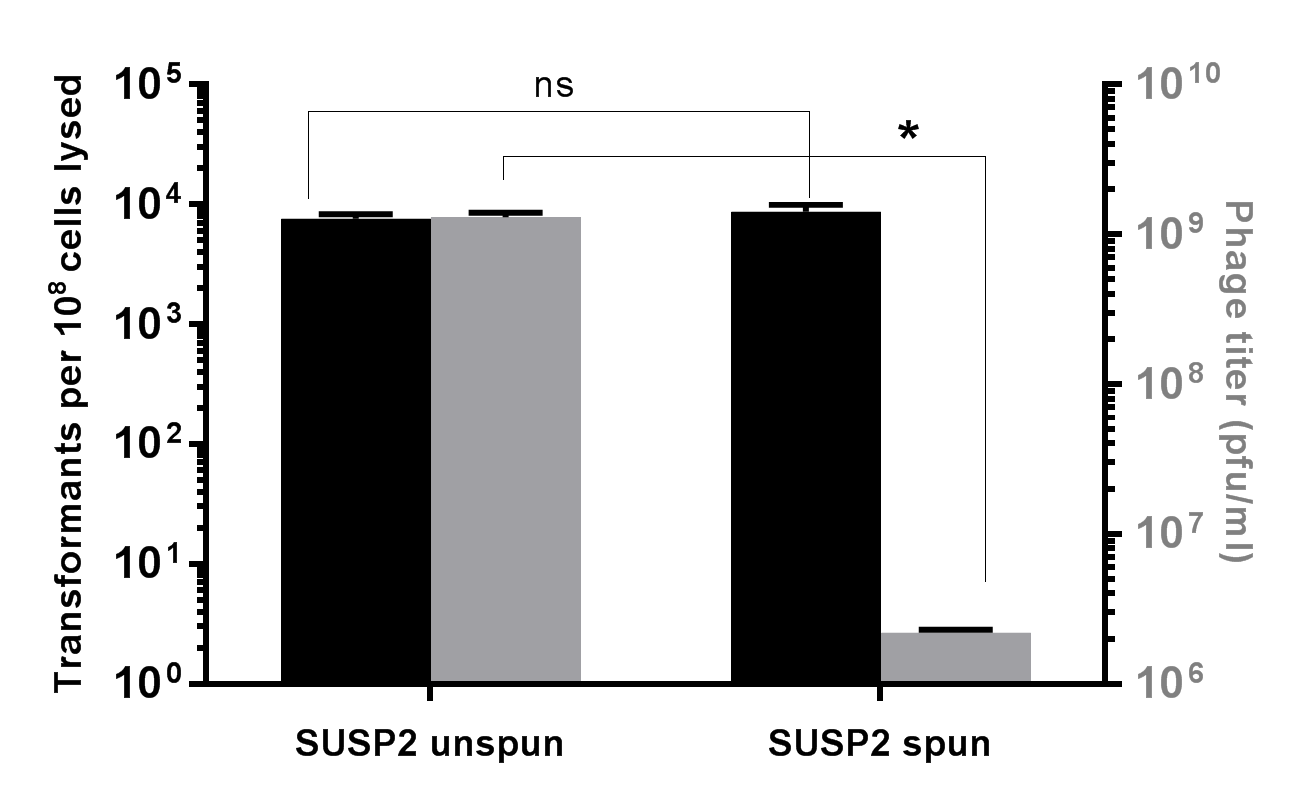

Supplement: FIGURE S1 [file mbo002173145sf1.tif]

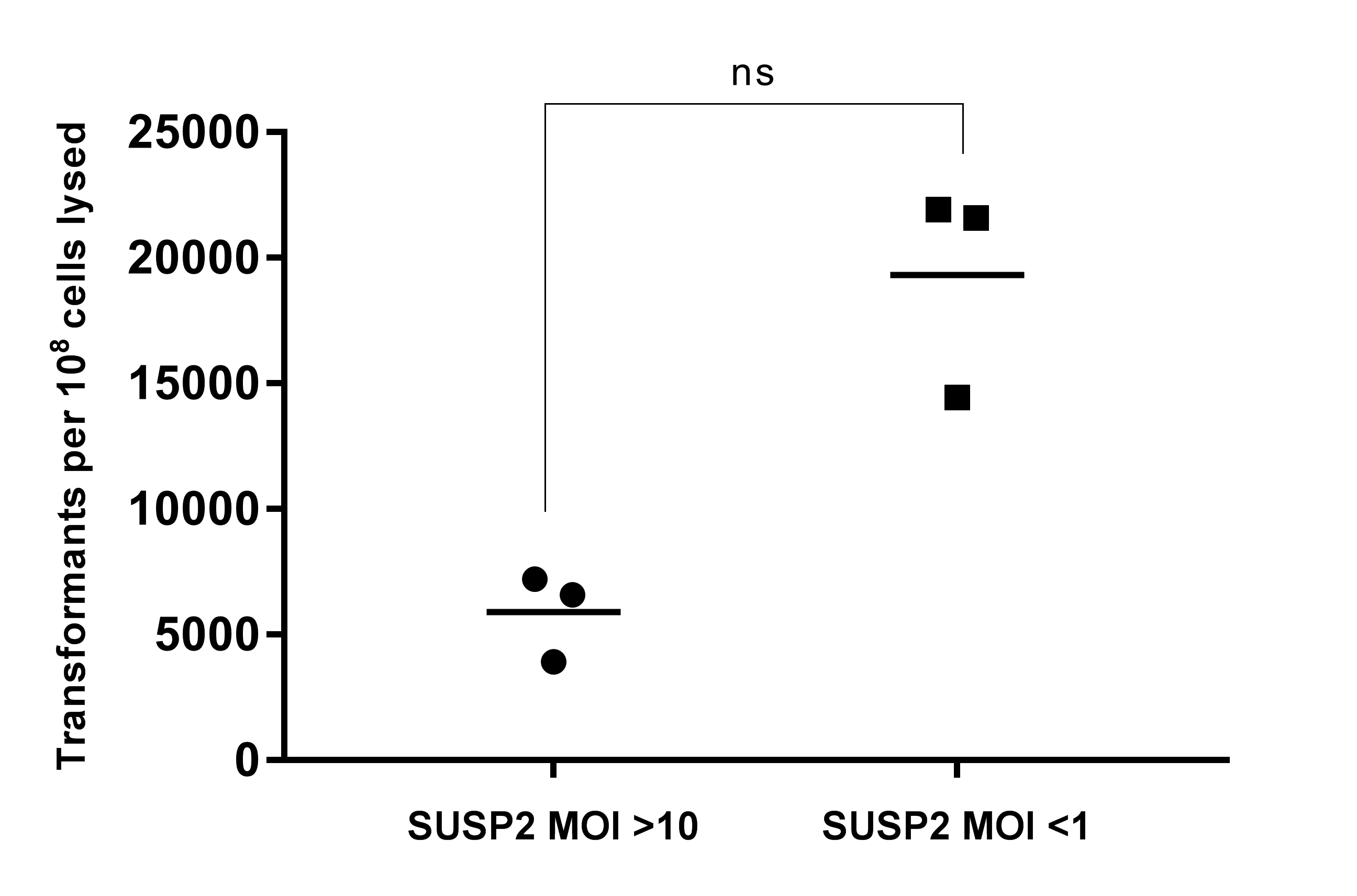

Supplement: FIGURE S2 [file mbo002173145sf2.tif]

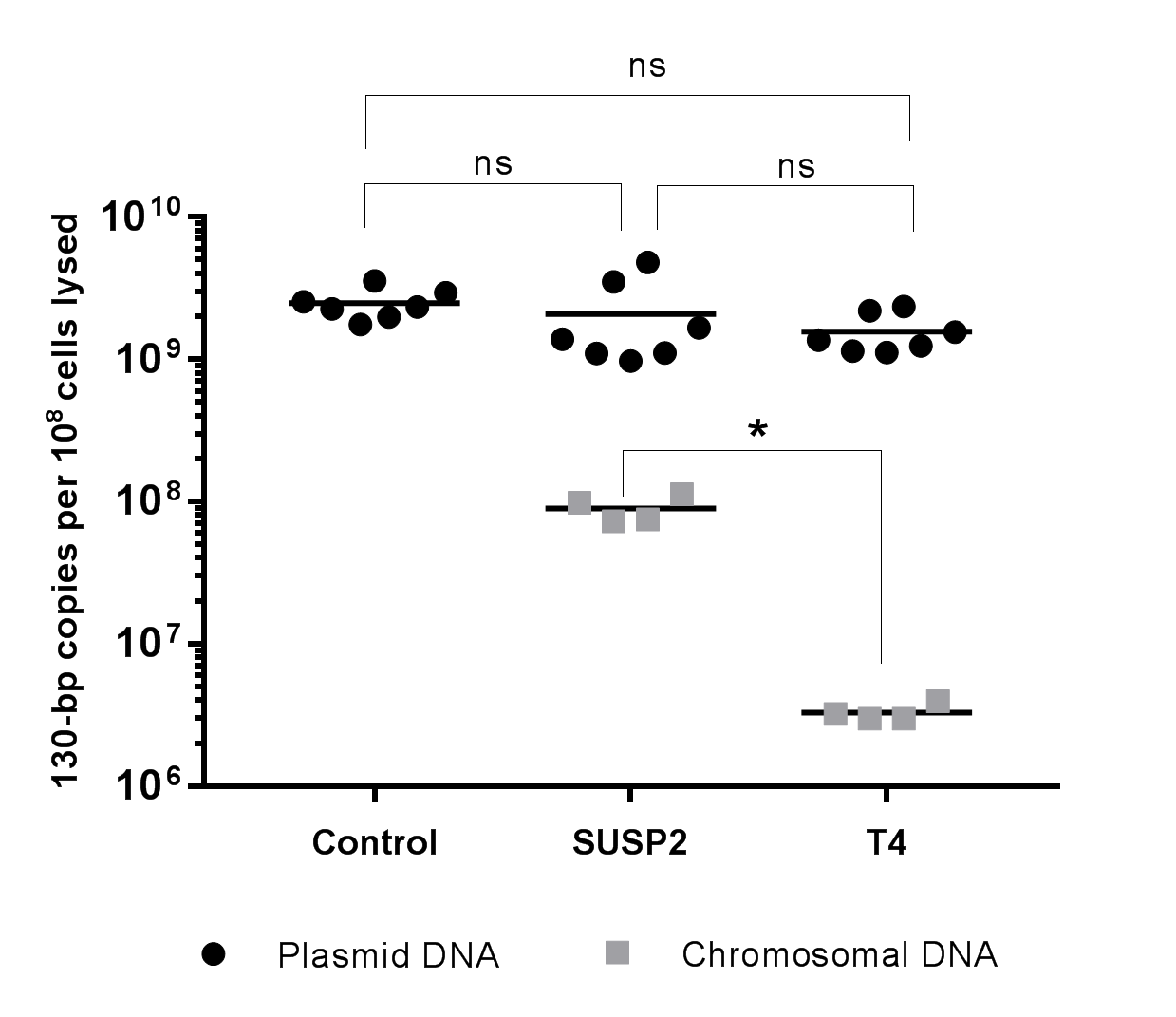

Supplement: FIGURE S3 [file mbo002173145sf3.tif]

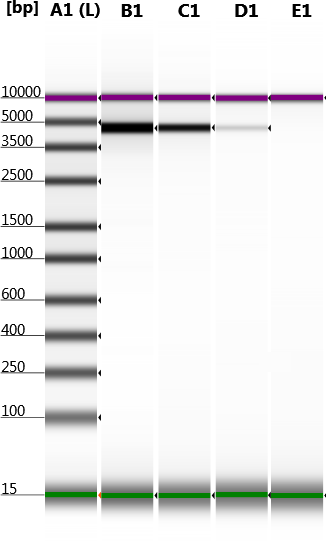

Supplement: FIGURE S4 [file mbo002173145sf4.tif]

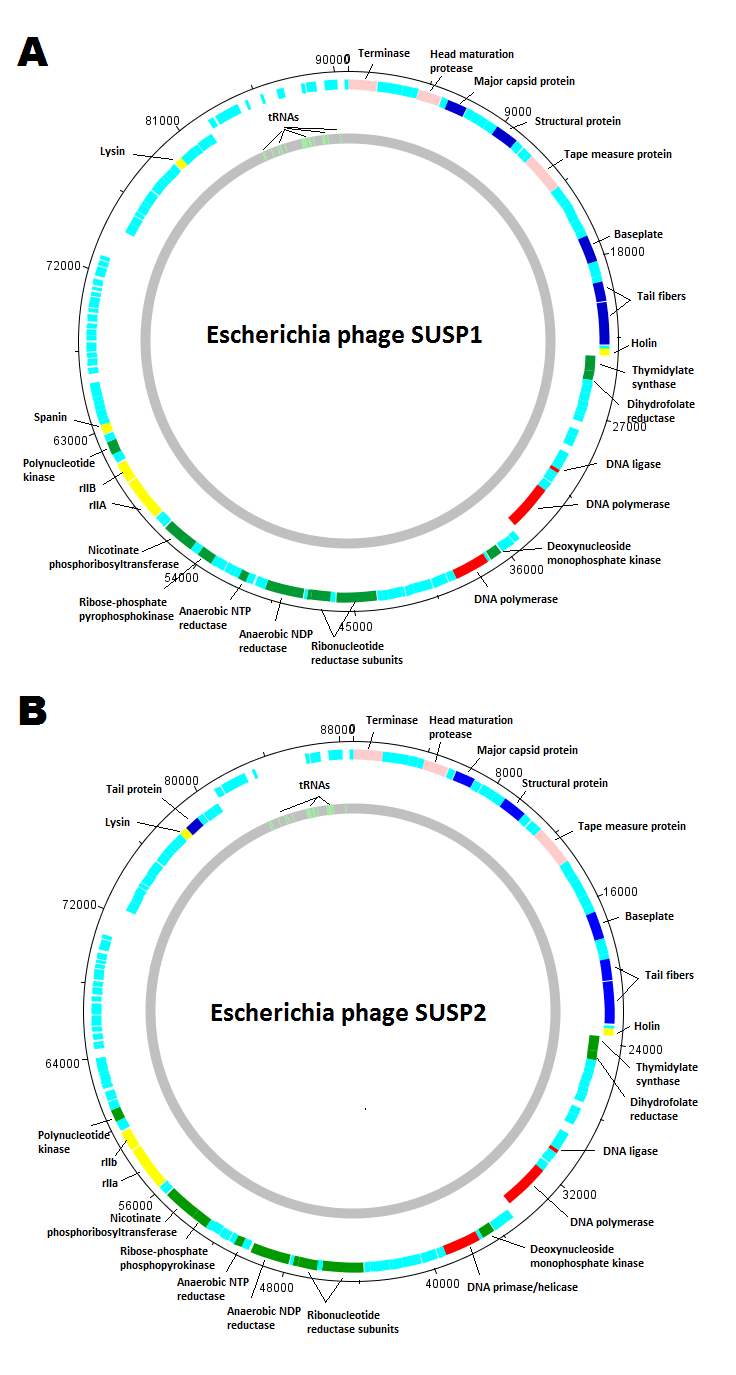

Supplement: FIGURE S5 [file mbo002173145sf5.tif]

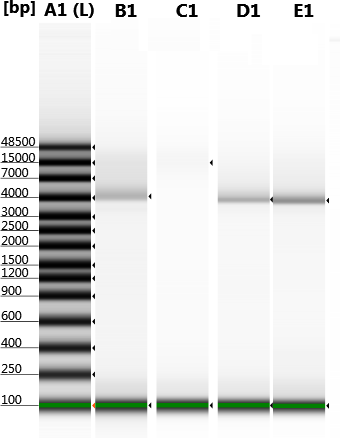

Supplement: FIGURE S7 [file mbo002173145sf7.tif]
