# Supplementary material for: Novel “Superspreader” Bacteriophages Promote Horizontal Gene Transfer by Transformation
Source: mBio. 2017 Jan 17;8(1):e02115-16. doi: 10.1128/mBio.02115-16 (PMC5241400; doi:10.1128/mBio.02115-16)
Supplement: TABLE S1 [file mbo002173145st1.docx]

**Supplementary Table 1**

**Table S1**. The 15 extant phage genome sequences most closely related to *Escherichia* phage SUSP1, as determined by whole-genome nucleotide BLAST (megablast) analysis.

| **Name** | **Accession** | **Max score** | **% identity** |
| --- | --- | --- | --- |
| [Escherichia phage SUSP2](http://blast.ncbi.nlm.nih.gov/Blast.cgi?CMD=Get&ALIGNDB_BATCH_ID=92698558&ALIGNDB_MASTER_ALIAS=SD_ALIGNDB_MASTER&ALIGNDB_MAX_ROWS=100&ALIGNDB_ORDER_CLAUSE=seq_evalue%20asc,aln_id%20asc&ALIGNDB_WHERE_CLAUSE=seq_evalue%20is%20not%20null&ALIGNMENTS=100&ALIGNMENT_VIEW=Pairwise&DATABASE_SORT=0&DESCRIPTIONS=100&DYNAMIC_FORMAT=on&FIRST_QUERY_NUM=0&FORMAT_OBJECT=Alignment&FORMAT_PAGE_TARGET=&FORMAT_TYPE=HTML&GET_SEQUENCE=yes&I_THRESH=&LINE_LENGTH=60&MASK_CHAR=2&MASK_COLOR=1&NUM_OVERVIEW=100&OLD_BLAST=false&PAGE=MegaBlast&QUERY_INDEX=0&QUERY_NUMBER=0&RESULTS_PAGE_TARGET=&RID=RUE1ZK6E01R&SHOW_LINKOUT=yes&SHOW_OVERVIEW=yes&STEP_NUMBER=&USE_ALIGNDB=true&OLD_VIEW=false&DISPLAY_SORT=1&HSP_SORT=1&CONFIG_DESCR=2,3,4,5,6,7,8#alnHdr_937264586) | [KT454806.1](http://www.ncbi.nlm.nih.gov/nucleotide/937264586?report=genbank&log$=nucltop&blast_rank=2&RID=RUE1ZK6E01R) | 54837 | 98% |
| [Escherichia phage JH2](http://blast.ncbi.nlm.nih.gov/Blast.cgi?CMD=Get&ALIGNDB_BATCH_ID=92698558&ALIGNDB_MASTER_ALIAS=SD_ALIGNDB_MASTER&ALIGNDB_MAX_ROWS=100&ALIGNDB_ORDER_CLAUSE=seq_evalue%20asc,aln_id%20asc&ALIGNDB_WHERE_CLAUSE=seq_evalue%20is%20not%20null&ALIGNMENTS=100&ALIGNMENT_VIEW=Pairwise&DATABASE_SORT=0&DESCRIPTIONS=100&DYNAMIC_FORMAT=on&FIRST_QUERY_NUM=0&FORMAT_OBJECT=Alignment&FORMAT_PAGE_TARGET=&FORMAT_TYPE=HTML&GET_SEQUENCE=yes&I_THRESH=&LINE_LENGTH=60&MASK_CHAR=2&MASK_COLOR=1&NUM_OVERVIEW=100&OLD_BLAST=false&PAGE=MegaBlast&QUERY_INDEX=0&QUERY_NUMBER=0&RESULTS_PAGE_TARGET=&RID=RUE1ZK6E01R&SHOW_LINKOUT=yes&SHOW_OVERVIEW=yes&STEP_NUMBER=&USE_ALIGNDB=true&OLD_VIEW=false&DISPLAY_SORT=1&HSP_SORT=1&CONFIG_DESCR=2,3,4,5,6,7,8#alnHdr_525336748) | [KF055347.1](http://www.ncbi.nlm.nih.gov/nucleotide/525336748?report=genbank&log$=nucltop&blast_rank=3&RID=RUE1ZK6E01R) | 22498 | 85% |
| [Salmonella phage HB-2014](http://blast.ncbi.nlm.nih.gov/Blast.cgi?CMD=Get&ALIGNDB_BATCH_ID=92698558&ALIGNDB_MASTER_ALIAS=SD_ALIGNDB_MASTER&ALIGNDB_MAX_ROWS=100&ALIGNDB_ORDER_CLAUSE=seq_evalue%20asc,aln_id%20asc&ALIGNDB_WHERE_CLAUSE=seq_evalue%20is%20not%20null&ALIGNMENTS=100&ALIGNMENT_VIEW=Pairwise&DATABASE_SORT=0&DESCRIPTIONS=100&DYNAMIC_FORMAT=on&FIRST_QUERY_NUM=0&FORMAT_OBJECT=Alignment&FORMAT_PAGE_TARGET=&FORMAT_TYPE=HTML&GET_SEQUENCE=yes&I_THRESH=&LINE_LENGTH=60&MASK_CHAR=2&MASK_COLOR=1&NUM_OVERVIEW=100&OLD_BLAST=false&PAGE=MegaBlast&QUERY_INDEX=0&QUERY_NUMBER=0&RESULTS_PAGE_TARGET=&RID=RUE1ZK6E01R&SHOW_LINKOUT=yes&SHOW_OVERVIEW=yes&STEP_NUMBER=&USE_ALIGNDB=true&OLD_VIEW=false&DISPLAY_SORT=1&HSP_SORT=1&CONFIG_DESCR=2,3,4,5,6,7,8#alnHdr_768214296) | [KP010413.1](http://www.ncbi.nlm.nih.gov/nucleotide/768214296?report=genbank&log$=nucltop&blast_rank=4&RID=RUE1ZK6E01R) | 22482 | 85% |
| [Escherichia phage EC6](http://blast.ncbi.nlm.nih.gov/Blast.cgi?CMD=Get&ALIGNDB_BATCH_ID=92698558&ALIGNDB_MASTER_ALIAS=SD_ALIGNDB_MASTER&ALIGNDB_MAX_ROWS=100&ALIGNDB_ORDER_CLAUSE=seq_evalue%20asc,aln_id%20asc&ALIGNDB_WHERE_CLAUSE=seq_evalue%20is%20not%20null&ALIGNMENTS=100&ALIGNMENT_VIEW=Pairwise&DATABASE_SORT=0&DESCRIPTIONS=100&DYNAMIC_FORMAT=on&FIRST_QUERY_NUM=0&FORMAT_OBJECT=Alignment&FORMAT_PAGE_TARGET=&FORMAT_TYPE=HTML&GET_SEQUENCE=yes&I_THRESH=&LINE_LENGTH=60&MASK_CHAR=2&MASK_COLOR=1&NUM_OVERVIEW=100&OLD_BLAST=false&PAGE=MegaBlast&QUERY_INDEX=0&QUERY_NUMBER=0&RESULTS_PAGE_TARGET=&RID=RUE1ZK6E01R&SHOW_LINKOUT=yes&SHOW_OVERVIEW=yes&STEP_NUMBER=&USE_ALIGNDB=true&OLD_VIEW=false&DISPLAY_SORT=1&HSP_SORT=1&CONFIG_DESCR=2,3,4,5,6,7,8#alnHdr_408384898) | [JX560968.1](http://www.ncbi.nlm.nih.gov/nucleotide/408384898?report=genbank&log$=nucltop&blast_rank=5&RID=RUE1ZK6E01R) | 22400 | 85% |
| [Salmonella phage Felix 01](http://blast.ncbi.nlm.nih.gov/Blast.cgi?CMD=Get&ALIGNDB_BATCH_ID=92698558&ALIGNDB_MASTER_ALIAS=SD_ALIGNDB_MASTER&ALIGNDB_MAX_ROWS=100&ALIGNDB_ORDER_CLAUSE=seq_evalue%20asc,aln_id%20asc&ALIGNDB_WHERE_CLAUSE=seq_evalue%20is%20not%20null&ALIGNMENTS=100&ALIGNMENT_VIEW=Pairwise&DATABASE_SORT=0&DESCRIPTIONS=100&DYNAMIC_FORMAT=on&FIRST_QUERY_NUM=0&FORMAT_OBJECT=Alignment&FORMAT_PAGE_TARGET=&FORMAT_TYPE=HTML&GET_SEQUENCE=yes&I_THRESH=&LINE_LENGTH=60&MASK_CHAR=2&MASK_COLOR=1&NUM_OVERVIEW=100&OLD_BLAST=false&PAGE=MegaBlast&QUERY_INDEX=0&QUERY_NUMBER=0&RESULTS_PAGE_TARGET=&RID=RUE1ZK6E01R&SHOW_LINKOUT=yes&SHOW_OVERVIEW=yes&STEP_NUMBER=&USE_ALIGNDB=true&OLD_VIEW=false&DISPLAY_SORT=1&HSP_SORT=1&CONFIG_DESCR=2,3,4,5,6,7,8#alnHdr_33340243) | [AF320576.1](http://www.ncbi.nlm.nih.gov/nucleotide/33340243?report=genbank&log$=nucltop&blast_rank=6&RID=RUE1ZK6E01R) | 22356 | 85% |
| [Salmonella phage SPT-1](http://blast.ncbi.nlm.nih.gov/Blast.cgi?CMD=Get&ALIGNDB_BATCH_ID=92698558&ALIGNDB_MASTER_ALIAS=SD_ALIGNDB_MASTER&ALIGNDB_MAX_ROWS=100&ALIGNDB_ORDER_CLAUSE=seq_evalue%20asc,aln_id%20asc&ALIGNDB_WHERE_CLAUSE=seq_evalue%20is%20not%20null&ALIGNMENTS=100&ALIGNMENT_VIEW=Pairwise&DATABASE_SORT=0&DESCRIPTIONS=100&DYNAMIC_FORMAT=on&FIRST_QUERY_NUM=0&FORMAT_OBJECT=Alignment&FORMAT_PAGE_TARGET=&FORMAT_TYPE=HTML&GET_SEQUENCE=yes&I_THRESH=&LINE_LENGTH=60&MASK_CHAR=2&MASK_COLOR=1&NUM_OVERVIEW=100&OLD_BLAST=false&PAGE=MegaBlast&QUERY_INDEX=0&QUERY_NUMBER=0&RESULTS_PAGE_TARGET=&RID=RUE1ZK6E01R&SHOW_LINKOUT=yes&SHOW_OVERVIEW=yes&STEP_NUMBER=&USE_ALIGNDB=true&OLD_VIEW=false&DISPLAY_SORT=1&HSP_SORT=1&CONFIG_DESCR=2,3,4,5,6,7,8#alnHdr_408386498) | [JX181822.1](http://www.ncbi.nlm.nih.gov/nucleotide/408386498?report=genbank&log$=nucltop&blast_rank=7&RID=RUE1ZK6E01R) | 22301 | 85% |
| [Salmonella phage FO1a](http://blast.ncbi.nlm.nih.gov/Blast.cgi?CMD=Get&ALIGNDB_BATCH_ID=92698558&ALIGNDB_MASTER_ALIAS=SD_ALIGNDB_MASTER&ALIGNDB_MAX_ROWS=100&ALIGNDB_ORDER_CLAUSE=seq_evalue%20asc,aln_id%20asc&ALIGNDB_WHERE_CLAUSE=seq_evalue%20is%20not%20null&ALIGNMENTS=100&ALIGNMENT_VIEW=Pairwise&DATABASE_SORT=0&DESCRIPTIONS=100&DYNAMIC_FORMAT=on&FIRST_QUERY_NUM=0&FORMAT_OBJECT=Alignment&FORMAT_PAGE_TARGET=&FORMAT_TYPE=HTML&GET_SEQUENCE=yes&I_THRESH=&LINE_LENGTH=60&MASK_CHAR=2&MASK_COLOR=1&NUM_OVERVIEW=100&OLD_BLAST=false&PAGE=MegaBlast&QUERY_INDEX=0&QUERY_NUMBER=0&RESULTS_PAGE_TARGET=&RID=RUE1ZK6E01R&SHOW_LINKOUT=yes&SHOW_OVERVIEW=yes&STEP_NUMBER=&USE_ALIGNDB=true&OLD_VIEW=false&DISPLAY_SORT=1&HSP_SORT=1&CONFIG_DESCR=2,3,4,5,6,7,8#alnHdr_347466883) | [JF461087.1](http://www.ncbi.nlm.nih.gov/nucleotide/347466883?report=genbank&log$=nucltop&blast_rank=8&RID=RUE1ZK6E01R) | 22275 | 85% |
| [Enterobacteriaphage UAB_Phi87](http://blast.ncbi.nlm.nih.gov/Blast.cgi?CMD=Get&ALIGNDB_BATCH_ID=92698558&ALIGNDB_MASTER_ALIAS=SD_ALIGNDB_MASTER&ALIGNDB_MAX_ROWS=100&ALIGNDB_ORDER_CLAUSE=seq_evalue%20asc,aln_id%20asc&ALIGNDB_WHERE_CLAUSE=seq_evalue%20is%20not%20null&ALIGNMENTS=100&ALIGNMENT_VIEW=Pairwise&DATABASE_SORT=0&DESCRIPTIONS=100&DYNAMIC_FORMAT=on&FIRST_QUERY_NUM=0&FORMAT_OBJECT=Alignment&FORMAT_PAGE_TARGET=&FORMAT_TYPE=HTML&GET_SEQUENCE=yes&I_THRESH=&LINE_LENGTH=60&MASK_CHAR=2&MASK_COLOR=1&NUM_OVERVIEW=100&OLD_BLAST=false&PAGE=MegaBlast&QUERY_INDEX=0&QUERY_NUMBER=0&RESULTS_PAGE_TARGET=&RID=RUE1ZK6E01R&SHOW_LINKOUT=yes&SHOW_OVERVIEW=yes&STEP_NUMBER=&USE_ALIGNDB=true&OLD_VIEW=false&DISPLAY_SORT=1&HSP_SORT=1&CONFIG_DESCR=2,3,4,5,6,7,8#alnHdr_402760815) | [JN225449.1](http://www.ncbi.nlm.nih.gov/nucleotide/402760815?report=genbank&log$=nucltop&blast_rank=9&RID=RUE1ZK6E01R) | 22022 | 85% |
| [Citrobacter phage Moogle](http://blast.ncbi.nlm.nih.gov/Blast.cgi?CMD=Get&ALIGNDB_BATCH_ID=92698558&ALIGNDB_MASTER_ALIAS=SD_ALIGNDB_MASTER&ALIGNDB_MAX_ROWS=100&ALIGNDB_ORDER_CLAUSE=seq_evalue%20asc,aln_id%20asc&ALIGNDB_WHERE_CLAUSE=seq_evalue%20is%20not%20null&ALIGNMENTS=100&ALIGNMENT_VIEW=Pairwise&DATABASE_SORT=0&DESCRIPTIONS=100&DYNAMIC_FORMAT=on&FIRST_QUERY_NUM=0&FORMAT_OBJECT=Alignment&FORMAT_PAGE_TARGET=&FORMAT_TYPE=HTML&GET_SEQUENCE=yes&I_THRESH=&LINE_LENGTH=60&MASK_CHAR=2&MASK_COLOR=1&NUM_OVERVIEW=100&OLD_BLAST=false&PAGE=MegaBlast&QUERY_INDEX=0&QUERY_NUMBER=0&RESULTS_PAGE_TARGET=&RID=RUE1ZK6E01R&SHOW_LINKOUT=yes&SHOW_OVERVIEW=yes&STEP_NUMBER=&USE_ALIGNDB=true&OLD_VIEW=false&DISPLAY_SORT=1&HSP_SORT=1&CONFIG_DESCR=2,3,4,5,6,7,8#alnHdr_701946626) | [KM236239.1](http://www.ncbi.nlm.nih.gov/nucleotide/701946626?report=genbank&log$=nucltop&blast_rank=10&RID=RUE1ZK6E01R) | 21605 | 85% |
| [Escherichia phage vB_EcoM_AYO145A](http://blast.ncbi.nlm.nih.gov/Blast.cgi?CMD=Get&ALIGNDB_BATCH_ID=92698558&ALIGNDB_MASTER_ALIAS=SD_ALIGNDB_MASTER&ALIGNDB_MAX_ROWS=100&ALIGNDB_ORDER_CLAUSE=seq_evalue%20asc,aln_id%20asc&ALIGNDB_WHERE_CLAUSE=seq_evalue%20is%20not%20null&ALIGNMENTS=100&ALIGNMENT_VIEW=Pairwise&DATABASE_SORT=0&DESCRIPTIONS=100&DYNAMIC_FORMAT=on&FIRST_QUERY_NUM=0&FORMAT_OBJECT=Alignment&FORMAT_PAGE_TARGET=&FORMAT_TYPE=HTML&GET_SEQUENCE=yes&I_THRESH=&LINE_LENGTH=60&MASK_CHAR=2&MASK_COLOR=1&NUM_OVERVIEW=100&OLD_BLAST=false&PAGE=MegaBlast&QUERY_INDEX=0&QUERY_NUMBER=0&RESULTS_PAGE_TARGET=&RID=RUE1ZK6E01R&SHOW_LINKOUT=yes&SHOW_OVERVIEW=yes&STEP_NUMBER=&USE_ALIGNDB=true&OLD_VIEW=false&DISPLAY_SORT=1&HSP_SORT=1&CONFIG_DESCR=2,3,4,5,6,7,8#alnHdr_806934097) | [KR014248.1](http://www.ncbi.nlm.nih.gov/nucleotide/806934097?report=genbank&log$=nucltop&blast_rank=11&RID=RUE1ZK6E01R) | 17634 | 88% |
| [Escherichia phage HY02](http://blast.ncbi.nlm.nih.gov/Blast.cgi?CMD=Get&ALIGNDB_BATCH_ID=391963572&ALIGNDB_MASTER_ALIAS=SD_ALIGNDB_MASTER&ALIGNDB_MAX_ROWS=100&ALIGNDB_ORDER_CLAUSE=seq_total_bit_score%20desc,aln_id%20asc&ALIGNDB_ORDER_CLAUSE=seq_bit_score%20desc,aln_id%20asc&ALIGNDB_WHERE_CLAUSE=seq_total_bit_score%20is%20not%20null&ALIGNMENTS=100&ALIGNMENT_VIEW=Pairwise&DATABASE_SORT=0&DESCRIPTIONS=100&DYNAMIC_FORMAT=on&FIRST_QUERY_NUM=0&FORMAT_OBJECT=Alignment&FORMAT_PAGE_TARGET=&FORMAT_TYPE=HTML&GET_SEQUENCE=yes&I_THRESH=&LINE_LENGTH=60&MASK_CHAR=2&MASK_COLOR=1&NUM_OVERVIEW=100&OLD_BLAST=false&PAGE=MegaBlast&QUERY_INDEX=0&QUERY_NUMBER=0&RESULTS_PAGE_TARGET=&RID=RU9XP6NC015&SHOW_LINKOUT=yes&SHOW_OVERVIEW=yes&STEP_NUMBER=&USE_ALIGNDB=true&WWW_BLAST_TYPE_URL=&OLD_VIEW=false&DISPLAY_SORT=1&HSP_SORT=1&CONFIG_DESCR=2,3,4,5,6,7,8#alnHdr_673939081) | [KM092515.1](http://www.ncbi.nlm.nih.gov/nucleotide/673939081?report=genbank&log$=nucltop&blast_rank=12&RID=RU9XP6NC015) | 17529 | 87% |
| Salmonella phage FSL SP-107 | [KC139640.1](http://www.ncbi.nlm.nih.gov/nucleotide/451939267?report=genbank&log$=nucltop&blast_rank=13&RID=RU9XP6NC015) | 17509 | 88% |
| Escherichia coli O157 typing phage 12 | [KP869110.1](http://www.ncbi.nlm.nih.gov/nucleotide/815008855?report=genbank&log$=nucltop&blast_rank=14&RID=RU9XP6NC015) | 17446 | 87% |
| [Escherichia coli O157 typing phage 11](http://blast.ncbi.nlm.nih.gov/Blast.cgi?CMD=Get&ALIGNDB_BATCH_ID=391963572&ALIGNDB_MASTER_ALIAS=SD_ALIGNDB_MASTER&ALIGNDB_MAX_ROWS=100&ALIGNDB_ORDER_CLAUSE=seq_total_bit_score%20desc,aln_id%20asc&ALIGNDB_ORDER_CLAUSE=seq_bit_score%20desc,aln_id%20asc&ALIGNDB_WHERE_CLAUSE=seq_total_bit_score%20is%20not%20null&ALIGNMENTS=100&ALIGNMENT_VIEW=Pairwise&DATABASE_SORT=0&DESCRIPTIONS=100&DYNAMIC_FORMAT=on&FIRST_QUERY_NUM=0&FORMAT_OBJECT=Alignment&FORMAT_PAGE_TARGET=&FORMAT_TYPE=HTML&GET_SEQUENCE=yes&I_THRESH=&LINE_LENGTH=60&MASK_CHAR=2&MASK_COLOR=1&NUM_OVERVIEW=100&OLD_BLAST=false&PAGE=MegaBlast&QUERY_INDEX=0&QUERY_NUMBER=0&RESULTS_PAGE_TARGET=&RID=RU9XP6NC015&SHOW_LINKOUT=yes&SHOW_OVERVIEW=yes&STEP_NUMBER=&USE_ALIGNDB=true&WWW_BLAST_TYPE_URL=&OLD_VIEW=false&DISPLAY_SORT=1&HSP_SORT=1&CONFIG_DESCR=2,3,4,5,6,7,8#alnHdr_815008722) | [KP869109.1](http://www.ncbi.nlm.nih.gov/nucleotide/815008722?report=genbank&log$=nucltop&blast_rank=15&RID=RU9XP6NC015) | 17446 | 87% |
| Enterobacteria phage wv8 | [EU877232.1](http://www.ncbi.nlm.nih.gov/nucleotide/216262944?report=genbank&log$=nucltop&blast_rank=17&RID=RU9XP6NC015) | 17444 | 87% |
