# Supplementary material for: Novel “Superspreader” Bacteriophages Promote Horizontal Gene Transfer by Transformation
Source: mBio. 2017 Jan 17;8(1):e02115-16. doi: 10.1128/mBio.02115-16 (PMC5241400; doi:10.1128/mBio.02115-16)
Supplement: TABLE S2 [file mbo002173145st2.docx]

**Supplementary Table 2**

**Table S2**. Descriptions of bacteria, phages, plasmids, and primers used in this study.

| **Name** | **Genotype** | | | **Source** |
| --- | --- | --- | --- | --- |
|  |  | | |  |
| *Escherichia coli* MG1655 | F-, λ-, *ilvG*-, *rfb*-, 50 *rph-1* | | | Lab stock |
| *Escherichia coli* Le392 | F-, *hsd*R514(rk–, mk+), *gln*V(supE44), *try*T (supF58), *lac*Y1 or Δ(lacIZY)6, *gal*K2, *gal*T22, *met*B1, *trp*R55 | | | N. Majdalani |
| λ*cI*^-^ | *att*^-^, *cI*^-^ | | | Lab stock |
| T4GT7 | amC87g42, amE51g56, rNB5060, alc | | | N. Majdalani |
| pBAD24 | pBR322 origin; ampicillin maker | | | Lab stock |
| pOAR31 | RSF1010 origin; kanamycin marker | | | D. Chattoraj |
| pSP102m5 | mini-P1 origin with copy-up mutation; chloramphenicol marker | | | D. Chattoraj |
| pπγ | R6K origin; kanamycin marker | | | D. Chattoraj |
|  |  | | |  |
| **Primer** | | **Direction** | **Function** | |
| 5’TGAAGATCAGTTGGGTGCAC | | Forward | ddPCR of plasmid pBAD24 fragment | |
| 5’CGCGCCACATAGCAGAACTT | | Reverse | ddPCR of plasmid pBAD24 fragment | |
|  | |  |  | |
| 5’CGTTATGGCAATCGTGGTCT | | Forward | ddPCR of *E. coli* *rpoS* gene fragment | |
| 5’GAATCCACCAGGTTGCGTAT | | Reverse | ddPCR of *E. coli* *rpoS* gene fragment | |
|  | |  |  | |
| 5’ACCCTATGCTACTCCGTCAA | | Forward | Amplification of whole plasmid pBAD24 | |
| 5’ACATGAGCAGATCCTCTACG | | Reverse | Amplification of whole plasmid pBAD24 | |
|  | |  |  | |
| 5’AGAGTTTGATCMTGGCTCAG | | Forward #1 | Sanger sequencing of soil isolate “WY10” | |
| 5’ATTACCGCGGCTGCTGG | | Reverse #1 | Sanger sequencing of soil isolate “WY10” | |
| 5’TAAAACTYAAAKGAATTGACGGG | | Forward #2 | Sanger sequencing of soil isolate “WY10” | |
| 5’CGGTTACCTTGTTACGACTT | | Reverse #2 | Sanger sequencing of soil isolate “WY10” | |
|  | |  |  | |
| 5’GACTATTAACAGCTCTTGC | | Forward | Tiling of SUSP1 (0-16 kb) | |
| 5’CAACGGCTTAATTGCCTC | | Reverse | Tiling of SUSP1 (0-16 kb) | |
| 5’GTCTCTTAAATCACCCGT | | Forward | Tiling of SUSP1 (12-28 kb) | |
| 5’CTGGTAACCCATCTGGAA | | Reverse | Tiling of SUSP1 (12-28 kb) | |
| 5’CAGACCACCTCCTTATGAA | | Forward | Tiling of SUSP1 (24-40 kb) | |
| 5’CCTTGTCTAATGGTGCTG | | Reverse | Tiling of SUSP1 (24-40 kb) | |
| 5’TCTACCCTTCTTAACAGCC | | Forward | Tiling of SUSP1 (36-52 kb) | |
| 5’GTATTCGTCGTGGTCAGT | | Reverse | Tiling of SUSP1 (36-52 kb) | |
| 5’CTAACTCTGAAGCCATACCC | | Forward | Tiling of SUSP1 (48-64 kb) | |
| 5’CAGCAGAAAGCACAAGACT | | Reverse | Tiling of SUSP1 (48-64 kb) | |
| 5’CTAAAGACCTCTTACACCCT | | Forward | Tiling of SUSP1 (60-76 kb) | |
| 5’GAGCAAACTCCACACCTTCA | | Reverse | Tiling of SUSP1 (60-76 kb) | |
| 5’TGTGAATACGACGACCAG | | Forward | Tiling of SUSP1 (72-90 kb) | |
| 5’GGTACTATCCACTTGTCT | | Reverse | Tiling of SUSP1 (72-90 kb) | |
|  | |  |  | |
| 5’TGACTTATGGCGGTTATGGT | | Forward | Tiling of SUSP2 (0-16 kb) | |
| 5’GCAATGGTACAAGCTTTC | | Reverse | Tiling of SUSP2 (0-16 kb) | |
| 5’GTATCTGTAAAGGTTGGGCT | | Forward | Tiling of SUSP2 (16-32 kb) | |
| 5’TTCCTGTAGCATACATCCCT | | Reverse | Tiling of SUSP2 (16-32 kb) | |
| 5’AGGGATGTATGCTACAGGAA | | Forward | Tiling of SUSP2 (32-48 kb) | |
| 5’ATTCCTGTCTAGCTCTCTTC | | Reverse | Tiling of SUSP2 (32-48 kb) | |
| 5’GCTAGACAGGAATCTCAAAA | | Forward | Tiling of SUSP2 (48-64 kb) | |
| 5’CATTATTCTTCCTCTGGG | | Reverse | Tiling of SUSP2 (48-64 kb) | |
| 5’CCCAGAGGAAGAATAATG | | Forward | Tiling of SUSP2 (64-80 kb) | |
| 5’TTCCCTGTTGGTCTCGTT | | Reverse | Tiling of SUSP2 (64-80 kb) | |
| 5’GGTCAGAGTTAACAAGAGT | | Forward | Tiling of SUSP2 (72-88 kb) | |
| 5’GAAGGATTCTGGGAATGG | | Reverse | Tiling of SUSP2 (72-88 kb) | |
